# The forgotten drought of 1765–1768: Reconstructing and re‐evaluating historical droughts in the British and Irish Isles

**DOI:** 10.1002/joc.6521

**Published:** 2020-02-25

**Authors:** Conor Murphy, Robert L. Wilby, Tom Matthews, Csaba Horvath, Arlene Crampsie, Francis Ludlow, Simon Noone, Jordan Brannigan, Jamie Hannaford, Robert McLeman, Eva Jobbova

**Affiliations:** ^1^ Irish Climate Analysis and Research UnitS (ICARUS), Department of Geography Maynooth University, Co. Kildare Maynooth Ireland; ^2^ Department of Geography and Environment Loughborough University Loughborough UK; ^3^ School of Geography University College Dublin Dublin Ireland; ^4^ Trinity Centre for Environmental Humanities, School of Histories and Humanities Trinity College Dublin Dublin Ireland; ^5^ Centre for Ecology & Hydrology Wallingford UK; ^6^ Department of Geography and Environmental Studies Wilfrid Laurier University Waterloo Ontario Canada

**Keywords:** documentary sources, England and Wales precipitation, historical drought, Ireland, Scotland, UK, water planning

## Abstract

Historical precipitation records are fundamental for the management of water resources, yet rainfall observations typically span 100–150 years at most, with considerable uncertainties surrounding earlier records. Here, we analyse some of the longest available precipitation records globally, for England and Wales, Scotland and Ireland. To assess the credibility of these records and extend them further back in time, we statistically reconstruct (using independent predictors) monthly precipitation series representing these regions for the period 1748–2000. By applying the Standardized Precipitation Index at 12‐month accumulations (SPI‐12) to the observed and our reconstructed series we re‐evaluate historical meteorological droughts. We find strong agreement between observed and reconstructed drought chronologies in post‐1870 records, but divergence in earlier series due to biases in early precipitation observations. Hence, the 1800s decade was less drought prone in our reconstructions relative to observations. Overall, the drought of 1834–1836 was the most intense SPI‐12 event in our reconstruction for England and Wales. Newspaper accounts and documentary sources confirm the extent of impacts across England in particular. We also identify a major, “forgotten” drought in 1765–1768 that affected the British‐Irish Isles. This was the most intense event in our reconstructions for Ireland and Scotland, and ranks first for accumulated deficits across all three regional series. Moreover, the 1765–1768 event was also the most extreme multi‐year drought across all regional series when considering 36‐month accumulations (SPI‐36). Newspaper and other sources confirm the occurrence and major socio‐economic impact of this drought, such as major rivers like the Shannon being fordable by foot. Our results provide new insights into historical droughts across the British Irish Isles. Given the importance of historical droughts for stress‐testing the resilience of water resources, drought plans and supply systems, the forgotten drought of 1765–1768 offers perhaps the most extreme benchmark scenario in more than 250‐years.

## Graphical abstract

We reconstruct and re‐evaluate historical droughts across the British Irish Isles (England and Wales, Scotland and Ireland) since 1748 and identify a major, “forgotten” drought in 1765–1768. We show that the event was likely the most extreme multi‐year drought across the region. Newspaper and other sources confirm the occurrence and major socio‐economic impact of this drought, which offers perhaps the most extreme benchmark scenario in more than 250‐years for stress‐testing drought and water resource plans.

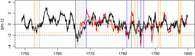

## 1 INTRODUCTION

Knowledge of historical meteorological droughts is important for understanding climate variability and change, contextualizing recent events (e.g., Kendon *et al*., 2013; Falzoi *et al*., 2018) and stress‐testing water supply systems by evaluating performance against past events (Watts *et al*., 2012; Wilby and Murphy, 2019). Water managers are especially interested in the ‘worst drought on record’ and ‘long droughts’ that persist for multiple seasons, given their impacts on surface and groundwater systems (e.g., Parry *et al*., 2012; Wilby *et al*., 2015). In England and Wales, the Environment Agency now requires water companies to ensure resilience to a drought event of 1 in 200 year severity (Environment Agency, 2017; Environment Agency, 2018), and the planning framework to 2065 highlights the importance of establishing ‘baseline drought risks’, and ‘worst historic droughts’ for assessing drought resilience (Water UK, 2016). Hence, data on historical droughts underpins such regulations and strategies and provides important input to condition stochastic analysis of extreme droughts (e.g., Serinaldi and Kilsby, 2012).

The British‐Irish Isles have some of the longest continuous observational series globally. For instance, Murphy *et al*. (2017) developed a 305‐year (1711–2016) monthly precipitation series for Ireland. In the UK the England and Wales Precipitation (EWP) series (Wigley *et al*., 1984; Wigley and Jones, 1987; Alexander and Jones, 2000; Simpson and Jones, 2014) provides a continuous monthly record from 1766, while Smith (1995) presents monthly precipitation for Scotland spanning 1757–1992.

Such long‐term precipitation series are critical for understanding historical meteorological droughts. Cole and Marsh (2006a) and Marsh *et al*. (2007) identified major droughts for England and Wales using a range of hydro‐meteorological data including EWP, temperature, river flows and groundwater for the period 1800–2006. They identified major drought events in 1798–1808, 1854–1860, 1887–1888, 1890–1909, 1921–1922, 1933–1934, 1959, 1976, 1990–1992 and 1995–1997. Furthermore, Cole and Marsh (2006a, 2006b) collated documentary evidence as a preliminary assessment of droughts and their impacts back to the 1700s, highlighting that the UK likely suffered from clusters of dry seasons or years during 1740–1744, 1780–1781, 1784–1786 and 1788–1789, with shorter droughts in 1705, 1736, 1765 and 1788.

These findings are largely consistent with those of Jones *et al*. (1997) who analysed droughts across the British‐Irish Isles since 1850. Similarly, Fowler and Kilsby (2002) examined historical droughts for Yorkshire from 1881 onwards. More recently, chronologies of streamflow droughts for the UK (Barker *et al*., 2019; Smith *et al*., 2019) from the 1890s has highlighted the expression of similar major droughts in hydrological terms, while other studies have shown how these events have manifested themselves in water supply systems (Watts *et al*., 2012; Lennard *et al*., 2016).

Some UK studies have identified drought events for individual long‐term precipitation records or sub‐regions of interest. For example, Todd *et al*. (2013) examined the severity, duration and frequency of drought in southeast England from 1697 to 2011 by applying the self‐calibrating Palmer Drought Severity Index (scPDSI) to observed data from Kew, Oxford and Spalding. In addition to the events identified by Cole and Marsh (2006a) and Marsh *et al*. (2007), they highlight less well documented droughts during eighteenth and nineteenth centuries, including; 1722–1726, 1730–1734, 1783–1791, 1801–1808, 1833–1836 and 1870–1872. They further note localized droughts in 1803 for Oxford, together with 1834 and 1835 for Spalding (Todd *et al*., 2013). Similarly, Spraggs *et al*. (2015) evaluated drought events for the east of the UK during 1798–2010 and concluded that the most severe events in 1854–1860 and 1893–1907 were characterized by contiguous dry winters and summers. Overall, they report that historical drought events for the Anglian region are consistent with Cole and Marsh (2006a) and Marsh *et al*. (2007), with the notable exception that the period around 1800 was much less severe.

For Ireland, Noone *et al*. (2017) derived a drought catalogue for the years 1765–2015 by applying the Standardized Precipitation Index (SPI‐12) to the island of Ireland precipitation network (1850–2015), together with an Irish composite series, derived as the average across the constituent stations. Prior to 1850, they estimated historical droughts using precipitation reconstructions representing the island as a whole. Noone *et al*. (2017) catalogue seven major drought rich periods for Ireland since 1850, namely; 1854–1860, 1884–1896, 1904–1912, 1921–1923, 1932–1935, 1952–1954 and 1969–1977. Droughts noted in the pre‐1850, reconstructed series were: 1784–1786, 1800–1804, 1805–1806, 1807–1809, 1813–1815, 1826–1827 and 1838–1839.

Wilby *et al*. (2015, 2016) examined the persistence of drought events across Britain and Ireland and found that the longest run of below average (1961–1990) rainfall since1870 persisted for 4 years in northern England and parts of Scotland during 1892–1896. For Ireland, the longest run of below average rainfall lasted up to 5 years regionally (in the east and southeast) and up to 4 years across the whole island during 1969–1973 (Wilby *et al*., 2016).

Although regional precipitation series have been central to understanding historical droughts, Murphy *et al*. (2020) identified biases in the early observed EWP series that are likely to affect other long‐term precipitation records for the British‐Irish Isles. Using independent indicators of surface pressure, atmospheric circulation and temperature, Murphy *et al*. (2020) reconstructed seasonal EWP and found that pre‐1870 winters in the observations are likely too dry due to under‐catch of snow. They also found that pre‐1820 observed EWP summer precipitation is likely too high, possibly due to uncertain data at key reference sites. Such biases potentially distort our understanding of historical droughts, and in particular multi‐season events associated with dry winters (Marsh *et al*., 2007; Spraggs *et al*., 2015).

Given these scientific and practical considerations, we statistically reconstruct monthly precipitation series for England and Wales, Scotland and Ireland using similar predictors to Murphy *et al*. (2020). We then apply the SPI to observed and reconstructed precipitation and re‐evaluate historical droughts. Finally, we use documentary evidence to corroborate our findings and to capture reported impacts of selected extreme events.

## 2 DATA AND METHODS

### 2.1 Regional precipitation series

We employ three long‐term regional precipitation series representing the British‐Irish Isles. For Ireland, we use the Island of Ireland 1711 (IoI_1711) (henceforth IoI) series which provides continuous monthly precipitation from 1711 to 2016 (Murphy *et al*., 2018). The post‐1850 series draws on quality assured monthly precipitation records derived by Noone *et al*. (2016), while the pre‐1850 series originates from instrumental and documentary sources compiled by the UK Met Office (Jenkinson *et al*., 1979a). Murphy *et al*. (2018) note that confidence in the early winter series of IoI_1711 is low prior to 1790, due to reliance on a small number of weather diaries, whereas summer precipitation for the latter half of the 1700s is likely too high, relative to other regional long‐term series. The monthly IoI series was accessed from PANGAEA (https://doi.pangaea.de/10.1594/PANGAEA.887593).

For the UK, we use two long‐term regional precipitation series. First, the EWP series (Wigley *et al*., 1984; Wigley and Jones, 1987; Alexander and Jones, 2000; Simpson and Jones, 2014) which is an area‐averaged precipitation series derived from five rainfall regions representing England and Wales. The series provides a continuous monthly precipitation record from 1766 and is routinely updated by the UK Met Office (UKMO) Hadley Centre, from whom monthly data were accessed (https://www.metoffice.gov.uk/hadobs/hadukp/).

Second, the monthly precipitation series for Scotland (1757–1992) (henceforth Scot) is an areal precipitation record based on UKMO data compiled from 1869 onwards, and extended to 1757 by Smith (1995) using unpublished UKMO data compiled by Jenkinson *et al*. (1979b). Smith (1995) corrected the series for known inhomogeneities. Between 1757 and 1765, Scottish precipitation is inferred from a single gauge at Carlisle (north Cumbria in England) and from 1766–1772 by two stations (Peebles in eastern Scotland, alongside Carlisle). No data are available for northern Scotland prior to 1820, hence the early series should be treated with caution (Smith, 1995).

### 2.2 Reconstruction of monthly precipitation

We statistically reconstructed each precipitation series using multiple linear regression. For each month, available long‐term observed Sea Level Pressure (SLP) and temperature data, together with a circulation index representing the North Atlantic Oscillation (NAO) were used as predictors. Each potential predictor variable is independent of the regional precipitation series and has been independently quality assured. Predictor selection and estimation of model coefficients were undertaken for the calibration period 1900–2000—a period of optimum overlap between the regional series and available predictors. Monthly simulations were evaluated for the years 1870–1899 and then used to reconstruct each series back to 1748 (EWP and IoI) and 1757 (Scot). The predictor variables employed were:

*Central England Temperature (CET)* (Manley, 1974; Parker *et al*., 1992). Previous research shows that CET winter and summer temperatures are strongly correlated with precipitation in the region (Murphy *et al*., 2018). Monthly CET data were downloaded from the UKMO (https://www.metoffice.gov.uk/hadobs/hadcet/).

*London Sea Level Pressure (LSLP)*: A 315‐year (1692–2007) daily series of mean SLP (MSLP) for the city of London (Cornes *et al*., 2012a; 2012b). Digitized data were transcribed from multiple sources, quality controlled, corrected and homogenized to represent daily means of MSLP at standard modern‐day conditions (Cornes *et al*., 2012a). Monthly values of MSLP are not reported when missing daily values exceed 20%. The record is less impacted by missing values from 1748 onwards and so we use LSLP from this point forward. Data were obtained from the Climatic Research Unit (CRU) at the University of East Anglia (https://crudata.uea.ac.uk/cru/data/parislondon/).

*Dublin (DSLP) and Edinburgh (ESLP) Sea Level Pressure*: monthly MSLP data for Dublin and Edinburgh were derived from the ADVICE database (Jones *et al*., 1987; Jones *et al*., 1999). The series for Dublin and Edinburgh cover the years 1831–2000 and 1770–1997, respectively. As reported by Jones *et al*. (1999), stations were subject to basic homogeneity checks to identify potential discrepancies in individual series by; (a) checking against grid based estimates of SLP for the years 1881–1995 and (b) comparing neighbouring stations over the available record. Data were accessed via the KNMI Climate Explorer. Given the strong correlation of London with Dublin (Pearson's *r* = .90) and Edinburgh (Pearson's *r* = .80) annual mean SLP, the LSLP series was used to extend both the DSLP and ESLP series to 1748 and 1757, respectively. Using linear regression, monthly adjustment factors (see Table S1) were derived from the overlapping period of record with LSLP and applied to extend both DSLP and ESLP. The same adjustment factors were used to update ESLP to 2000.

*Paris London Index (PL)*: An indicator of the state of the NAO index (NAOI) over the years 1,692–2007, providing a consistent monthly measure of westerly airflow over northwest Europe (Cornes *et al*., 2013). The index was developed from MSLP data recovered and corrected for the respective cities. Data were obtained from CRU (https://crudata.uea.ac.uk/cru/data/parislondon/).

A square root transform was applied before model selection if observed precipitation deviated from a normal distribution. For each month during the calibration period, the best model was identified using stepwise selection. Checks were made to ensure selected predictors were a statistically significant (*p* < .05) addition to the model and unaffected by multicollinearity, as indicated by the Variable Inflation Factor. Separate models containing DSLP, ESLP and LSLP, respectively, were derived for IoI, Scot and EWP. Table S2 lists the predictors selected and model performance for each month. The effect of regression parameter uncertainty in the calibration dataset on reconstructions was explored by fitting selected models using 1,000 bootstrap re‐samples of 50 years (with replacement) from the calibration dataset. Re‐samples were used to establish a median simulation for each month, together with 95% confidence intervals for model simulations. All models were assessed to ensure *iid* assumptions were met. Reconstructions for each month were compiled to derive a continuous monthly reconstructed series representing the regional series. The original precipitation series, together with 1,000 reconstructions of each regional series were used to re‐evaluate historical droughts.

### 2.3 Standardized precipitation index (SPI) and drought identification

The SPI (McKee *et al*., 1993; Guttman, 1999) was used to identify drought events in observed and reconstructed series. SPI is recommended by the World Meteorological Organization (2012) as a key drought indicator and only requires monthly precipitation as input. The index is calculated by summing precipitation over specified accumulation periods then fitting these moving totals to a parametric distribution, from which probabilities are transformed to the standard normal distribution (McKee *et al*., 1993; Guttman, 1999; Lloyd‐Hughes and Saunders, 2002). The resultant values give standard deviations from typical accumulated precipitation for a given location and time of year. SPI values between 0.99 and −0.99 are regarded as near normal, −1.00 to −1.49 as moderate drought, −1.50 to −1.99 as severe drought, and less than −2.00 as extreme drought (World Meteorological Organization, 2012).

The primary focus of our analysis is on the SPI for 12 month accumulations (SPI‐12). This block length is widely considered as indicative of hydrological drought (World Meteorological Organization, 2012), and is more likely to represent droughts with impacts across multiple sectors (Noone *et al*., 2016). However, we also derived SPI‐36 to capture multi‐year droughts of interest to water resource planners, particularly in parts of the British Irish Isles vulnerable to multi‐year rainfall deficiencies, due to groundwater storage, for example, south‐east England (Folland *et al*. 2015).

Derivation of SPI values was undertaken using the SPEI package in R (Beguería and Vicente‐Serrano, 2013). Here, SPI values were derived relative to the reference period 1900–2000, so that comparisons can easily be drawn between observed and model reconstructed series. Following Stagge *et al*. (2015), the two‐parameter gamma distribution was used to derive SPI values for each month. We derived SPI separately for the observed and each of the 1,000 model simulations, from which the median, maximum and minimum SPI series were compiled.

Individual drought events were identified from the three observed regional series and their equivalent median reconstructed SPI‐12 series. Drought onset was defined as the month in which SPI‐12 falls below −1.00, with a return to positive values indicating termination (Mishra and Singh, 2010; Lennard *et al*., 2016; Noone *et al*., 2016). Duration was calculated as the number of months from drought onset to termination, whilst the accumulated and mean deficits were derived as the sum of SPI‐12 over the drought event, and the sum of SPI‐12 over the drought event divided by duration, respectively. Finally, the maximum intensity of each drought was characterized by the minimum SPI‐12 value reached during the event in question.

### 2.4 Verifying drought detection

Following Rudd *et al*. (2017), a quantitative assessment of drought detection skill from the reconstructed precipitation series was undertaken for the period 1870–2000, given relative confidence in the observed data quality for these periods. Skill scores comprised the probability of detection (POD) and the false alarm ratio (FAR). As outlined by Rudd *et al*. (2017), if *h* is the number of droughts that are both observed and modelled (hits), and *m* is the number of observed droughts that are not modelled (misses), then *POD = h/(h + m)*. If *f* is the number of droughts that are modelled and not observed (false alarms) then *FAR = f/(h + f)*. POD and FAR range between zero and one with higher/lower values representing better skill, respectively. In addition, taking just drought hits, the POD of correct drought magnitude was derived by categorizing observed and reconstructed droughts as severe or moderate using the threshold of SPI < −1.5.

### 2.5 Documentary and other sources

We use documentary sources to further investigate selected drought events. In particular, we make use of digitized and searchable newspaper archives including the Irish (www.irishnewsarchive.com) and British Newspaper Archives (https://www.britishnewspaperarchive.co.uk/). Both resources provide coverage from the 1700s. These are supplemented with searches of a number of other resources including; ‘The Statistical Account of Scotland: Drawn up from the Communication of the Ministers of the Different Parishes, Volume 12’ (Sinclair, 1794); The ‘Climate of England or A Guide to the Knowledge of the Atmospheric Phenomena of England’ (Whistlecraft, 1840); ‘Rural Gleanings or Facts worth Knowing’ (Whistlecraft, 1851); and the 1851 Census of Ireland (Wilde, 1851).

## 3 RESULTS

### 3.1 Reconstruction of monthly precipitation

Selected models for each month, their correlation with observations for the calibration (1900–2000) and validation (1870–1899) periods, together with outcomes of tests for model assumptions are presented in Table S2. Reconstructions of EWP monthly precipitation with strongest correlations (*r*) between observed and median reconstructed (median of 1,000 bootstrapped simulations) precipitation during the calibration period ranged from *r =* .81 for July to *r* = .91 for November, December and February. IoI monthly reconstructions tend to be most weakly correlated with observations, with median scores ranging from *r* = .72 in July to *r* = .85 in February. All models transfer well to the validation period, returning similar correlation scores to calibration. Across each regional series, some monthly models have a greater proportion of simulations with non‐constant variance and non‐normal residuals than expected by chance. However, these have negligible effect on median model reconstructions, when models that violate assumptions are removed. All models were therefore retained.

**Figure 1:**
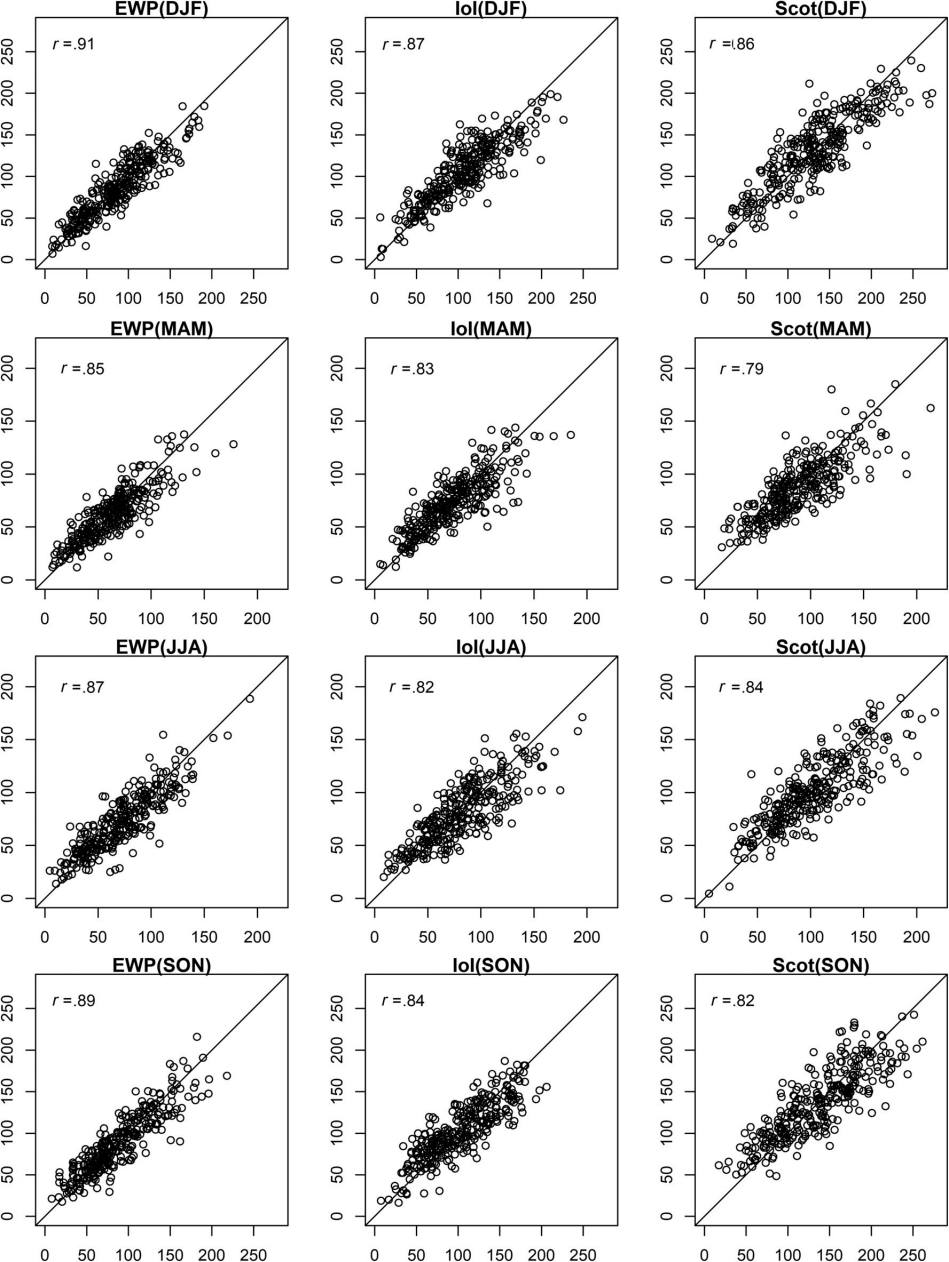
Observed (*x*‐axis) and median reconstructed (*y*‐axis) precipitation (mm) for EWP (left), Ireland (middle) and Scotland (right) for winter (DJF), spring (MAM), summer (JJA) and autumn (SON) months for the calibration period (1900–2000). Pearson's correlation coefficient and the 1:1 line are also shown

**Figure 2:**
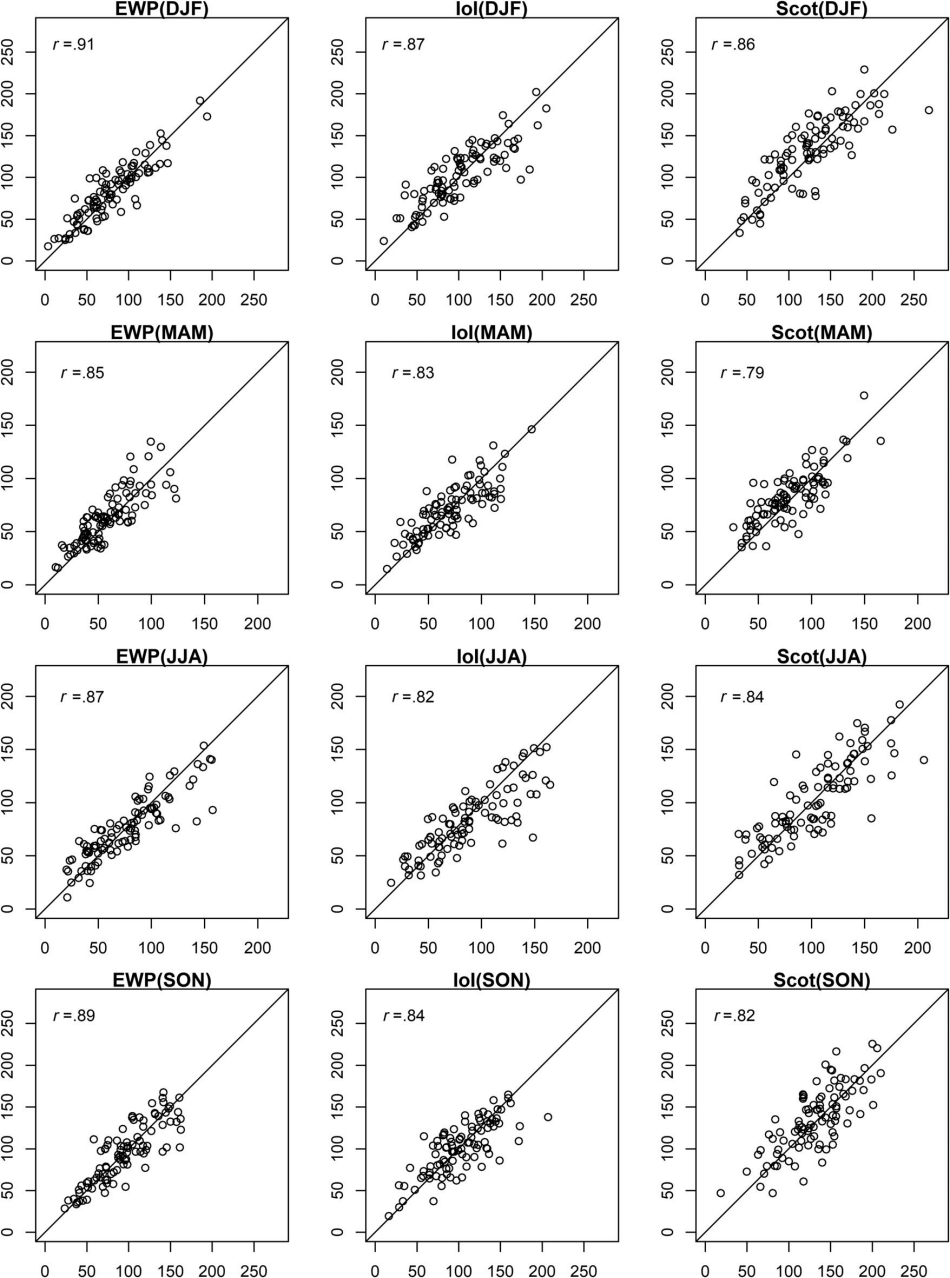
As Figure 1 but for the validation period (1870–1899)

Figures 1 and 2 show scatter plots of observed and median reconstructed monthly precipitation totals by season for EWP, IoI, and Scot for both calibration and validation periods, together with correlation scores. Reconstructions show good agreement with observations for each regional precipitation series for both calibration and validation. There is some evidence of increasing variance of the residuals for higher precipitation totals, however, this is unlikely to impact the assessment of drought conditions. In addition, there is a tendency for the driest months to be overestimated in our reconstructions for certain seasons in different precipitation series during calibration (e.g., spring and autumn months in Scot), which may be due to under‐catch of snowfall. However, there is limited evidence for such over‐estimation during the validation period.

**Figure 3:**
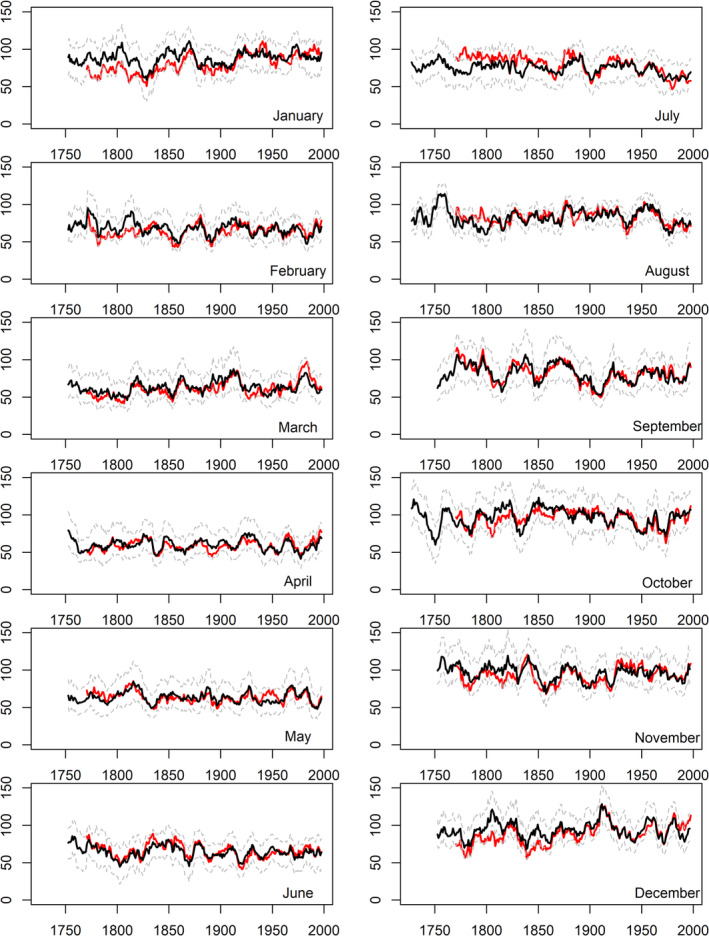
Observed (red) and reconstructed (black) decadal mean monthly precipitation totals (mm) for EWP. Dashed grey lines represent the 90% confidence interval of model reconstructions based on 1,000 bootstrap samples of the calibration data (see Section 2)

**Figure 4:**
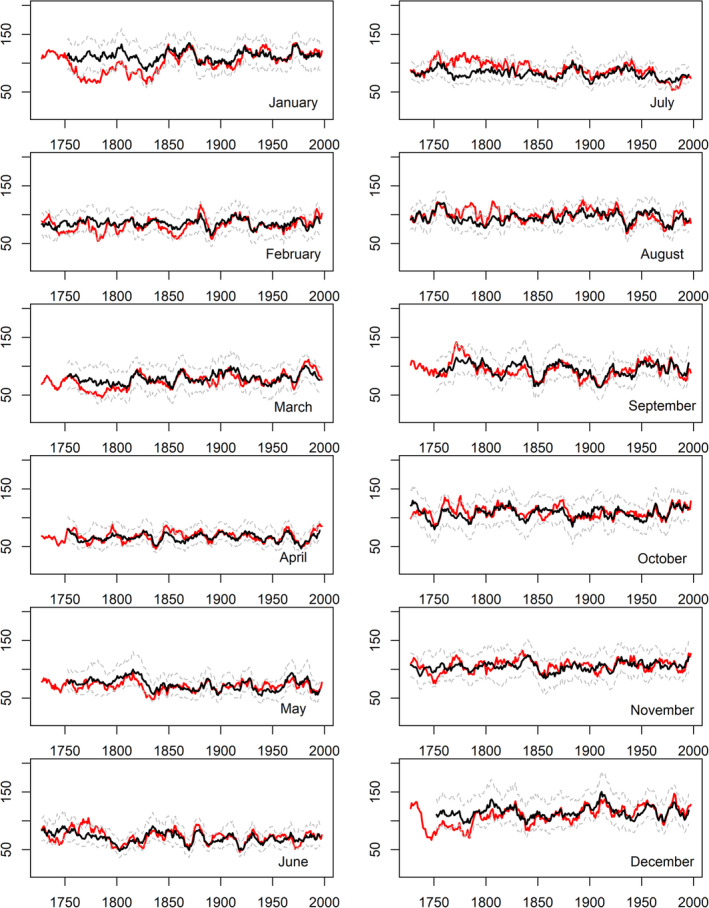
As in Figure 3 but for Island of Ireland (IoI) precipitation (mm)

**Figure 5:**
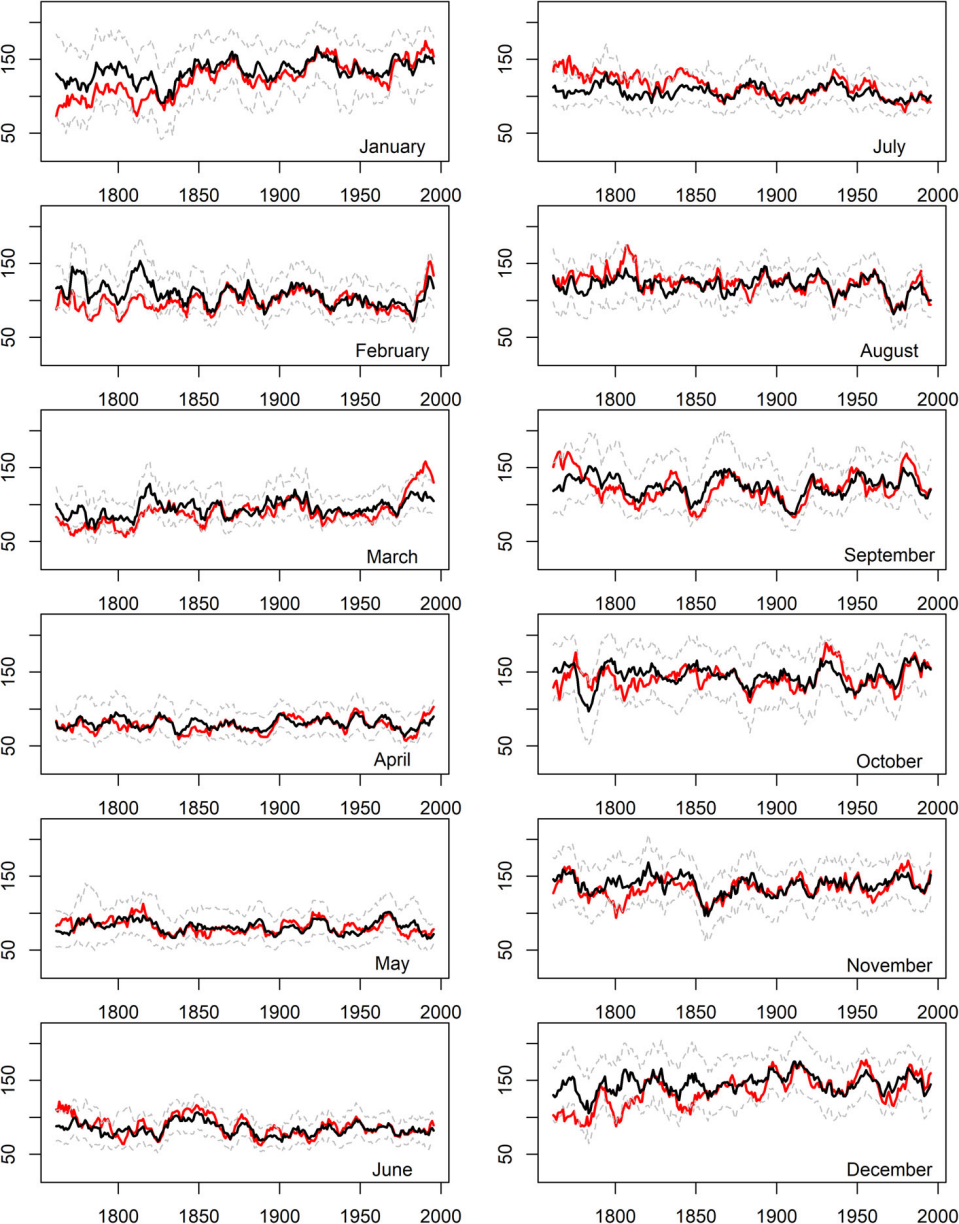
As in Figure 3 but for Scotland (Scot) precipitation (mm)

Figures 3, 4, 5 show rolling decadal monthly observed and reconstructed precipitation totals for EWP, IoI and Scot, respectively. It is evident that observed precipitation totals for winter [DJF] months, and at times March and October, in the pre‐1870 records tend to be too low relative to the reconstructions. In summer, July and August observed precipitation totals for the pre‐1850 record are too high relative to the reconstructions for all regional series, as reported in Murphy *et al*. (2020). For all other months there is close agreement between model reconstructions and observations.

### 3.2 Observed and reconstructed SPI‐12

SPI‐12 series were derived for observed and median reconstructed regional precipitation series. The POD of drought events in the median reconstructed EWP series is very high. Over the period 1870–2000, POD is 0.85, rising to 0.96 for the period 1900–2000, while the FAR is 0.15 for both periods. The POD for correct drought magnitude (moderate vs. severe, or SPI‐12 greater than or less than −1.5) for the median EWP reconstruction is 0.89 for 1870–2000 and 0.95 for 1900–2000. For the reconstructed IoI and Scot series, the POD skill is lower, but remains satisfactory. For IoI, the POD (FAR) of drought events is 0.69 (0.24) and 0.70 (0.27) for 1870–2000 and 1900–2000, respectively, while the POD for correct drought magnitude is 0.68 for both periods. Finally, for Scotland the POD (FAR) of drought events is 0.74 (0.24) and 0.70 (0.27) for 1870–2000 and 1900–2000, respectively. The POD for correct drought magnitude is 0.77 for 1870–2000 and 0.89 for 1900–2000.

### 3.3 EWP observed and reconstructed droughts 1748–2000

**Figure 6:**
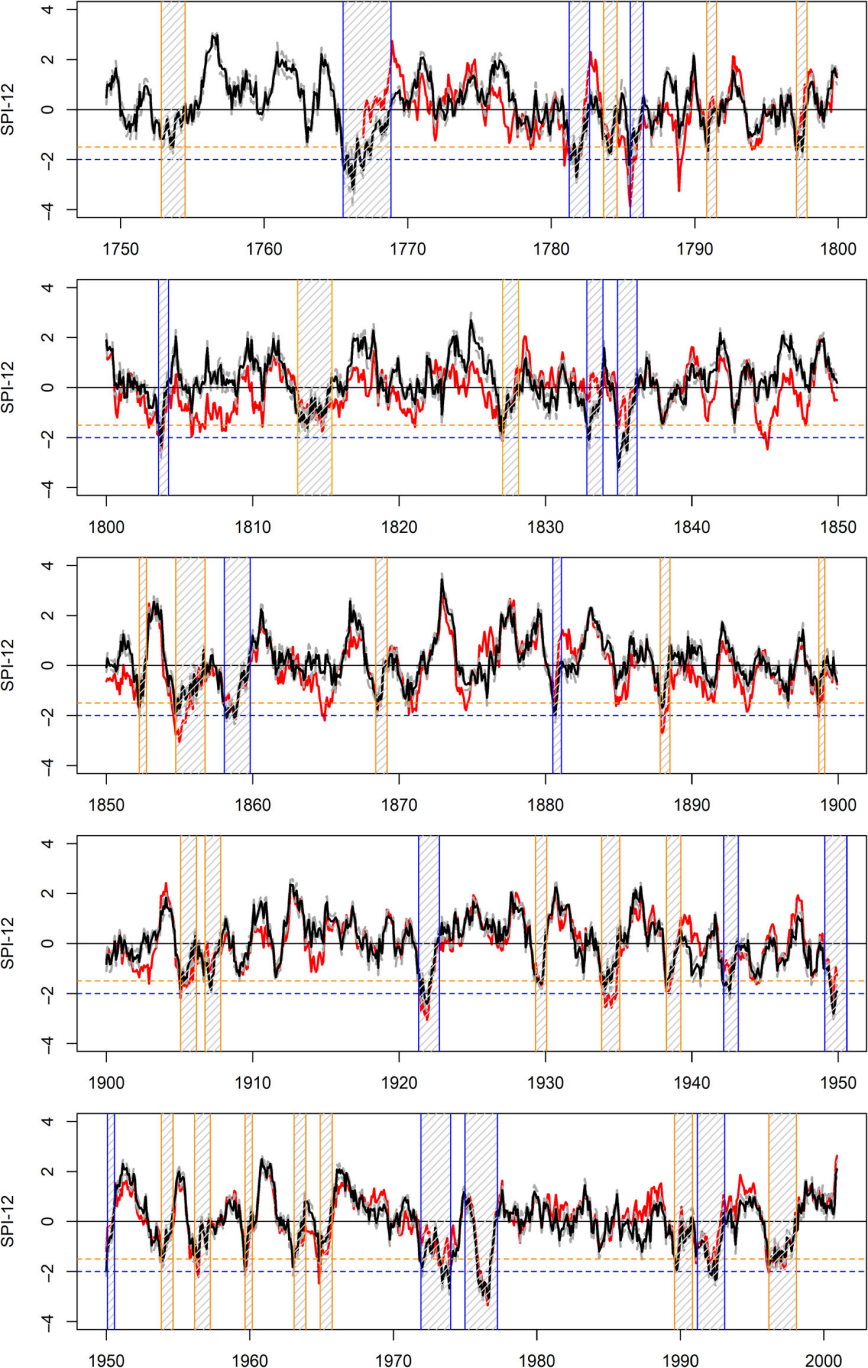
Observed (red) and median reconstructed (black) SPI‐12 series for EWP. Each panel shows an approximate 50 years block. Shaded areas are severe (orange) and extreme (blue) drought events. The dashed orange horizontal line shows the SPI‐12 threshold for classification as severe drought, while the dashed blue horizontal line shows the threshold for classification as extreme drought. Dashed grey lines represent the 90% confidence intervals for the reconstructed SPI‐12 series

Details for all droughts identified in the observed and median reconstructed SPI‐12 series of each regional precipitation record, together with associated drought statistics are provided in Tables S3‐S8. Figure 6 shows the observed and reconstructed SPI‐12 series for EWP, together with severe (orange) and extreme (blue) droughts identified from the median reconstruction. There is strong agreement between observed and median reconstructed SPI‐12 and associated drought events in the post 1870 period, with some minor exceptions. Extreme droughts in the observed EWP series in 1964–1965, 1933–1934 and 1887–1889 are classed more modestly as severe droughts in the reconstruction, with shorter duration. Additionally, the severe drought in observed EWP during 1896–1897 is classed as a moderate drought in reconstructions.

Prior to 1870 there is greater disagreement between the observed and reconstructed drought events. As with precipitation, the observed SPI‐12 series is generally too low relative to the reconstructions, meaning that many severe and extreme droughts in the observations are not present in the reconstructions. Of particular note are the 1850s, which in observed EWP is marked by two drought events around the middle and end of the decade. While the latter event is well captured by the reconstructions, the earlier event is classed as severe, rather than extreme. Extreme drought events in observed EWP in 1844–1846 and 1788–1789 are not even classed as droughts in the reconstructions. In the first decade of the 1800s, while the extreme drought of 1803–1804 is present in both observations and reconstructions, the remainder of the decade has normal or wet conditions according to reconstructed SPI‐12, yet persistent drought conditions are noted in the observed EWP series throughout this decade. Previous work by Cole and Marsh (2006a, 2006b) highlight the lack of documentary evidence for prolonged drought in this period, while Spraggs *et al*. (2015) note that the decade was less drought rich in their reconstructions of Anglian precipitation (see Section 4).

For the pre‐1870 series, three extreme drought events are identified in the reconstructions that are either not in the observations or occur prior to the commencement of EWP in 1766. These are 1832–1833, 1834–1836 and 1765–1768. The 1832–1833 drought commenced in October 1832 and ended in November 1833, lasting 12 months, with a minimum SPI‐12 of −2.15 and accumulated deficit of −14.33. The drought of 1834–1836 is present as a severe drought in the observed EWP series (min SPI‐12‐1.84), but is the most intense drought (minimum SPI‐12: −3.29) in the entire reconstructed EWP series. It also lasts longer (16 months) and has a greater accumulated deficit (−28.41) in reconstructions than observations.

However, the drought of 1765–1768 is most noteworthy. This drought, which ran from June 1765 to October 1768, commenced just before and overlaps the beginning of the observed EWP series. The event lasted 40 months and had a minimum SPI‐12 of −3.19. Notably, it stands apart from any other drought in either the observed or reconstructed EWP SPI‐12 series, with an accumulated deficit of −60.98. The earliest drought in the reconstructed EWP SPI‐12 series occurs between October 1752 and June 1754. Lowe (1870) refers to the river Tweed in northern England/southern Scotland as being “dried up” in February 1753.

Table 1 provides the top ten ranked drought events by accumulated deficit, duration and intensity (min SPI‐12) for observed and reconstructed SPI‐12 series for each regional precipitation series. The 1765–1768 event ranks first by accumulated deficit in the reconstructed EWP SPI‐12 series by a considerable margin, followed by 1971–1974 (−47.59). The same ordering is found for drought duration with 1765–1768 (40 months) followed by 1971–1974 (34 months). After 1834–1836 (−3.29), 1765–1768 ranks second (−3.19) and 1975–1977 ranks third (−3.09) as the most intense drought events in our reconstructions.

**Table 1:** Top 10 ranked drought events from observed and median reconstructed (.med) England Wales Precipitation (EWP), island of Ireland (IoI) and Scottish (Scot) SPI‐12 series. Droughts are ranked by accumulated deficit (Acc. Def.) (top), duration (dur.) in months (middle) and intensity (inten.) or minimum SPI‐12 (bottom)

	EWP		EWP.med		IoI		IoI.med		Scot		Scot.med	
	*Years*	*Acc. def*.	*Years*	*Acc. def*.	*Years*	*Acc. def*.	*Years*	*Acc. def*.	*Years*	*Acc. def*.	*Years*	*Acc. def*.
*Accumulated deficit*	1784–1787	−50.57	1765–1768	−60.97	1854–1860	−100.84	1765–1768	−57.00	1800–1807	−149.24	1765–1769	−86.34
	1805–1809	−46.99	1971–1974	−47.59	1784–1787	−57.93	1971–1974	−38.92	1853–1859	−100.19	1780–1784	−76.55
	1854–1856	−46.22	1975–1977	−32.87	1905–1908	−42.82	1813–1815	−33.17	1885–1890	−94.80	1854–1859	−67.41
	1996–1998	−33.48	1991–1993	−31.19	1971–1974	−41.81	1857–1859	−33.12	1783–1787	−90.86	1971–1974	−39.24
	1933–1935	−32.05	1834–1836	−28.41	1748–1751	−41.61	1952–1954	−30.12	1971–1974	−55.36	1939–1941	−37.75
	1975–1977	−31.37	1857–1859	−28.03	1932–1934	−37.99	1905–1907	−29.37	1765–1767	−50.74	1879–1882	−36.22
	1942–1945	−30.10	1996–1998	−27.77	1842–1846	−37.00	1955–1958	−29.23	1813–1815	−50.18	1812–1815	−33.14
	1780–1782	−29.66	1813–1815	−26.35	1765–1768	−35.93	1783–1786	−27.47	1824–1827	−47.58	1996–1998	−30.80
	1921–1922	−29.19	1949–1950	−25.70	1975–1977	−32.03	1939–1941	−24.30	1879–1882	−42.18	1975–1977	−30.32
	1844–1846	−28.48	1854–1856	−24.98	1788–1789	−31.08	1975–1977	−23.50	1869–1872	−41.23	1785–1787	−27.80

	*Years*	*Dur*.	*Years*	*Dur*.	*Years*	*Dur*.	*Years*	*Dur*.	*Years*	*Dur*.	*Years*	*Dur*.
*Duration (months)*	1784–1787	42	1765–1768	40	1854–1860	73	1765–1768	42	1800–1807	85	1854–59	53
	1805–1809	40	1971–1974	34	1842–1846	41	1971–1974	34	1885–1890	64	1765–1769	47
	1942–1945	35	1813–1815	28	1784–1787	37	1783–1786	33	1853–1859	63	1780–1784	47
	1854–1856	29	1854–1856	24	1905–1908	36	1905–1907	32	1783–1787	55	1971–1974	34
	1814–1816	29	1991–1993	23	1971–1974	36	1944–1946	31	1895–1898	45	1812–1815	27
	1898–1900	29	1996–1998	23	1748–1751	29	1813–1815	29	1824–1827	40	1879–1882	26
	1972–1974	26	1857–1859	22	1765–1768	28	1955–1958	27	1971–1974	39	1785–1787	26
	1996–1998	24	1752–1754	20	1924–1923	28	1857–1859	26	1963–1965	33	1996–1998	23
	1904–1906	24	1949–1950	18	1932–1934	25	1854–1856	24	1869–1872	31	1939–1941	22
	1864–1866	24	1781–1782	17	1800–1802	24	1837–1839	23	1879–1882	28	1870–1872	22

	*Years*	*Inten*.	*Years*	*Inten*.	*Years*	*Inten*.	*Years*	*Inten*.	*Years*	*Inten*.	*Years*	*Inten*.
*Intensity (min SPI‐12)*	1784–1787	−3.86	1834–1836	−3.29	1784–1787	−4.84	1765–1768	−3.14	1783–1787	−5.38	1765–1769	−4.45
	1975–1977	−3.35	1765–1768	−3.19	1788–1789	−4.12	1851–1852	−2.93	1765–1767	−4.92	1780–1784	−3.58
	1788–1789	−3.26	1975–1977	−3.09	1932–1934	−3.31	1971–1974	−2.61	1813–1815	−4.09	1939–1941	−3.08
	1854–1856	−3.06	1949–1950	−2.80	1765–1768	−3.27	1887–1888	−2.59	1796–1797	−3.71	1879–1882	−2.96
	1921–1922	−3.05	1781–1782	−2.69	1887–1886	−3.23	1975–1977	−2.51	1788–1789	−3.58	1975–1977	−2.90
	1780–1782	−2.91	1971–1974	−2.67	1826–1828	−3.08	1955–1958	−2.49	1780–1782	−3.55	1762–1763	−2.79
	1802–1804	−2.73	1921–1922	−2.42	1796–1798	−3.06	1952–1954	−2.38	1800–1807	−3.19	1829–1831	−2.73
	1887–1889	−2.73	1991–1993	−2.37	1854–1860	−2.98	1959–1960	−2.28	1959–1960	−3.16	1941–1943	−2.64
	1933–1935	−2.57	1803–1804	−2.31	1824–1825	−2.82	1857–1859	−2.25	1879–1882	−3.16	1864–1866	−2.57
	1964–1965	−2.48	1785–1786	−2.22	1803–1804	−2.80	1880–1881	−2.24	1762–1763	−3.09	1812–1815	−2.43

### 3.4 IoI observed and reconstructed droughts 1748–2000

**Figure 7:**
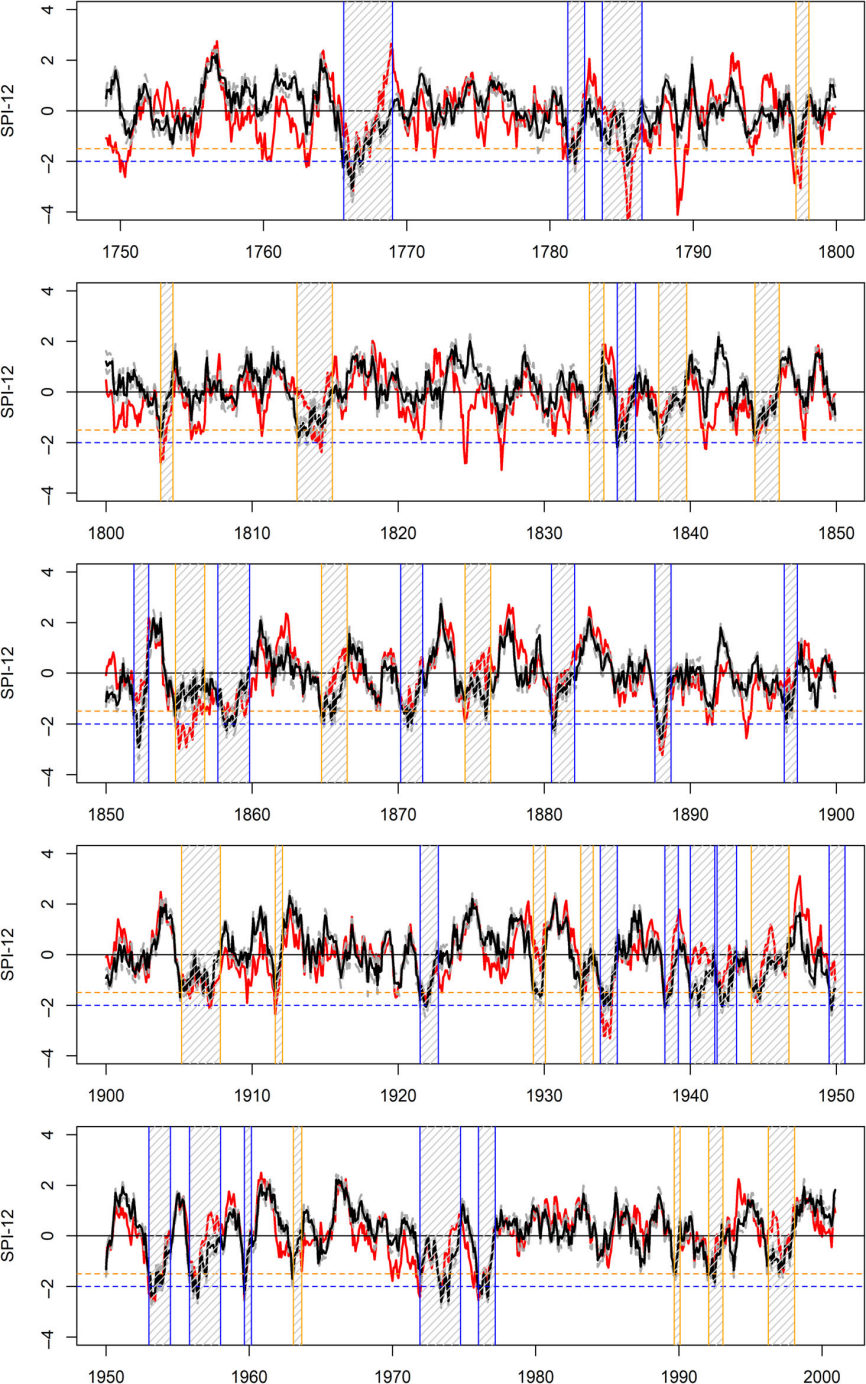
As in Figure 6 but for Island of Ireland (IoI)

Figure 7 compares observed and median reconstructed IoI SPI‐12 series (with severe and extreme droughts in the reconstructed series highlighted). As with EWP, there is strong coherence between observed and simulated events post 1870, with greater divergence in the pre‐1870 record. Droughts in the early 1970s, 1933–1934 and 1911–1912 and the 1890s are, however, not as extreme in our reconstructions compared with observations. In the pre‐1870 record, some notable differences emerge. The reconstructed 1850s event is marked by a severe drought in the early part of the decade, a return to moderate drought conditions during the middle of the decade, before another extreme drought at the end of the decade.

In the pre‐1850 record, the IoI precipitation series is based on fewer gauged observations and relies on weather diaries. For this period, many extreme droughts in the observations are not evident in our reconstructions, and thus assumed to be due to early data biases. As with EWP, the persistent drought conditions in the 1800s decade are not evident in our reconstructions for IoI, though the years 1803–1804 do contain a severe drought in our reconstructions. Extreme droughts are identified in the reconstructions for 1834–1836, 1783–1786, 1781–1782 and 1765–1768. Both the 1834–1836 and 1765–1768 events are also evident in the observed series. By far the most notable event in the entire reconstructed series is the 1765–1768 drought which ranks first in terms of accumulated deficit (−57.00), duration (42 months) and intensity (min SPI‐12‐3.14; see Table 1).

### 3.5 Scot observed and reconstructed droughts 1757–2000

**Figure 8:**
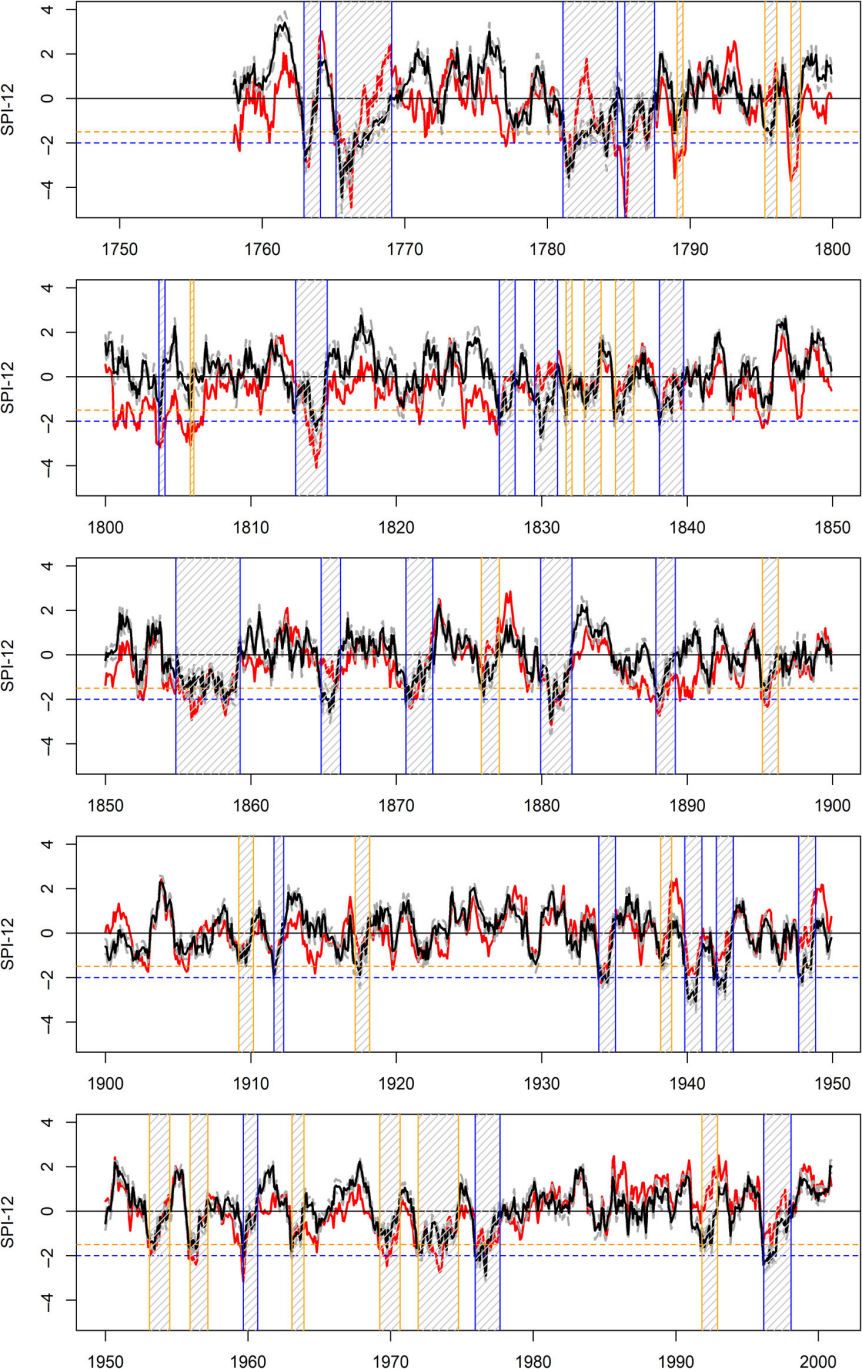
As in Figure 6 but for Scotland (Scot)

Observed and reconstructed SPI‐12 series for Scotland, together with associated severe and extreme droughts for the latter, are shown in Figure 8. During 1870–2000 there is again strong agreement between observed and reconstructed SPI‐12 series. However, some divergence is evident in the classification of droughts as severe or extreme. For instance, the 1971–1974 drought is classed as severe in reconstructions, but extreme in the observations. On the other hand, droughts during the 1940s are classed as extreme in the reconstructions, but severe or moderate in the observed series.

As the number of gauges contributing to the Scottish precipitation series decreases in the early records, the degree of divergence between observed and reconstructed SPI‐12 increases, particularly pre‐1830. As with EWP and IoI, the first decade of the 1800s is not nearly as drought rich in our reconstructions as in observations. Two short droughts are identified in 1803–1804 (severe) and 1805–1806 (extreme), whereas the remainder of the decade shows normal to wet conditions. Similarly, extreme droughts in the late 1780s and 1790s in the observations, are only present as severe or moderate droughts in the reconstructions.

Of note from the Scottish SPI‐12 reconstructions are the protracted extreme droughts, in excess of 40 months duration, in the early records. These include 1854–1859 (53 months), 1780–1784 (47 months) and 1765–1769 (47 months). Each of these events are also evident in the observations, but tend to be more moderate in the reconstructions. These episodes surpass any other droughts since 1860 in terms of duration and accumulated deficit (Table 1). Indeed, the 1765–1769 event ranks first in the entire record in terms of accumulated deficit (−86.34), and intensity (min SPI‐12: −4.45), and second in terms of duration to the 1854–1859 drought. Other notable extreme droughts in the early records include 1812–1815 (27 months) and 1785–1787 (26 months). The 1834–1836 drought (identified as most intense in EWP) is also present in the Scottish reconstructions, though classed as severe (min SPI‐12‐1.89).

### 3.6 SPI‐36 long duration droughts

**Figure 9:**
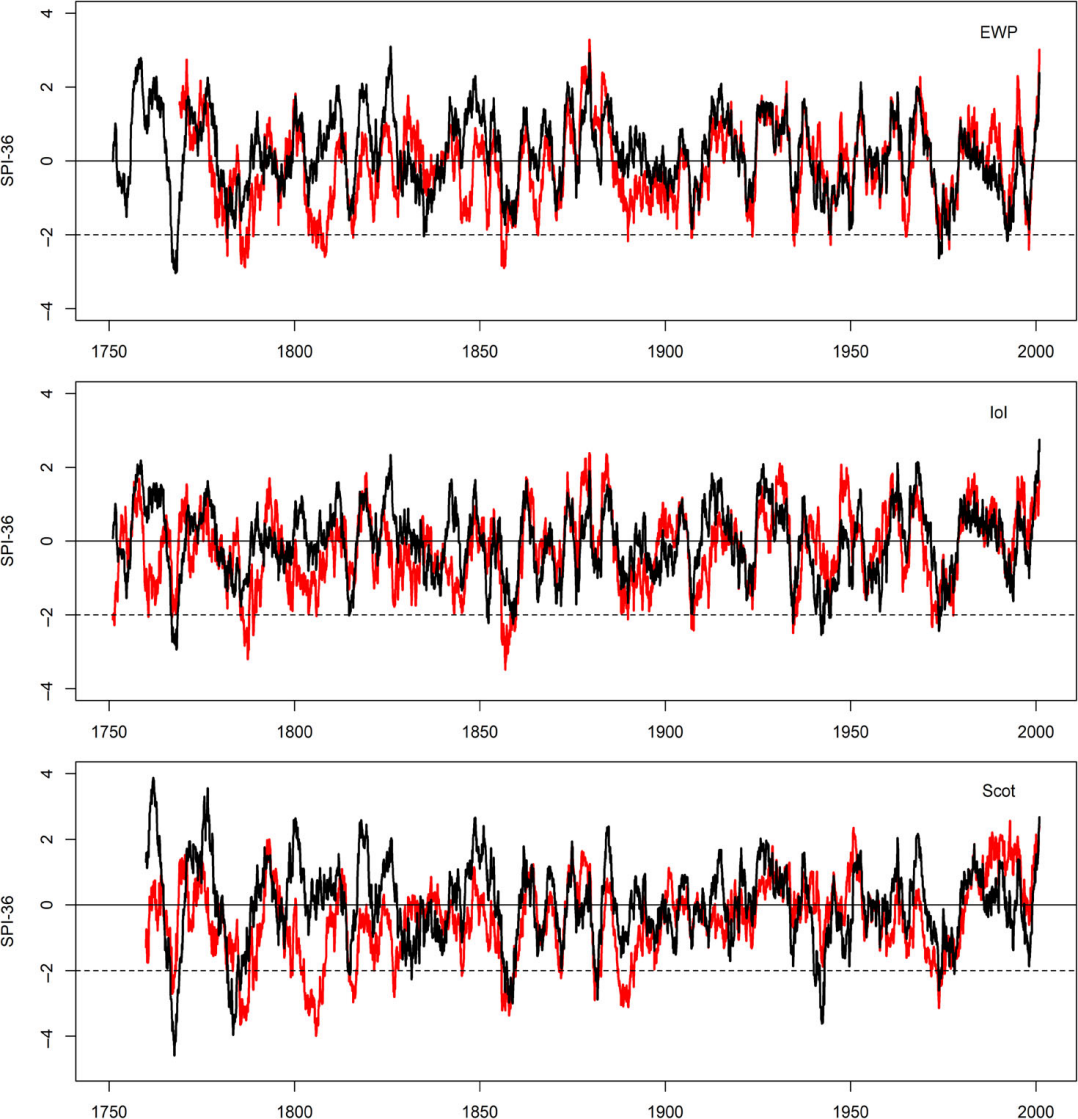
Observed (red) and median reconstructed (black) SPI‐36 for EWP (top), IoI (middle) and Scot (bottom). The dashed horizontal line represents the threshold for classification as extreme drought

Although SPI‐12 is representative of hydrological droughts, many groundwater dependent systems are vulnerable to multi‐year deficits, especially in southeast England. Therefore, we also briefly examine 3‐year accumulated deficits (SPI‐36) for each reconstructed series. Figure 9 shows SPI‐36 for observed and median reconstructed SPI series for each regional series. For EWP, IoI and Scot, the 1765 drought stands out as the most extreme drought in the entire reconstructed series. Additionally, in each reconstructed series the observed drought conditions of the 1800s are not present in the reconstructions, while drought during the 1850s in EWP and IoI are less severe than observed.

### 3.7 Documentary evidence for the 1765–1768 and 1834–1836 droughts

Droughts in 1765–1768 and 1834–1836 stand out as major events that have been poorly documented in the literature. Therefore, we turn to newspaper archives and additional documentary sources to build further confidence in our reconstructions and to shed light on the socio‐economic impact of these events. Links to specific documentary sources mentioned are provided in Supporting Information.

#### 3.7.1 The 1765–1768 drought

**Figure 10:**
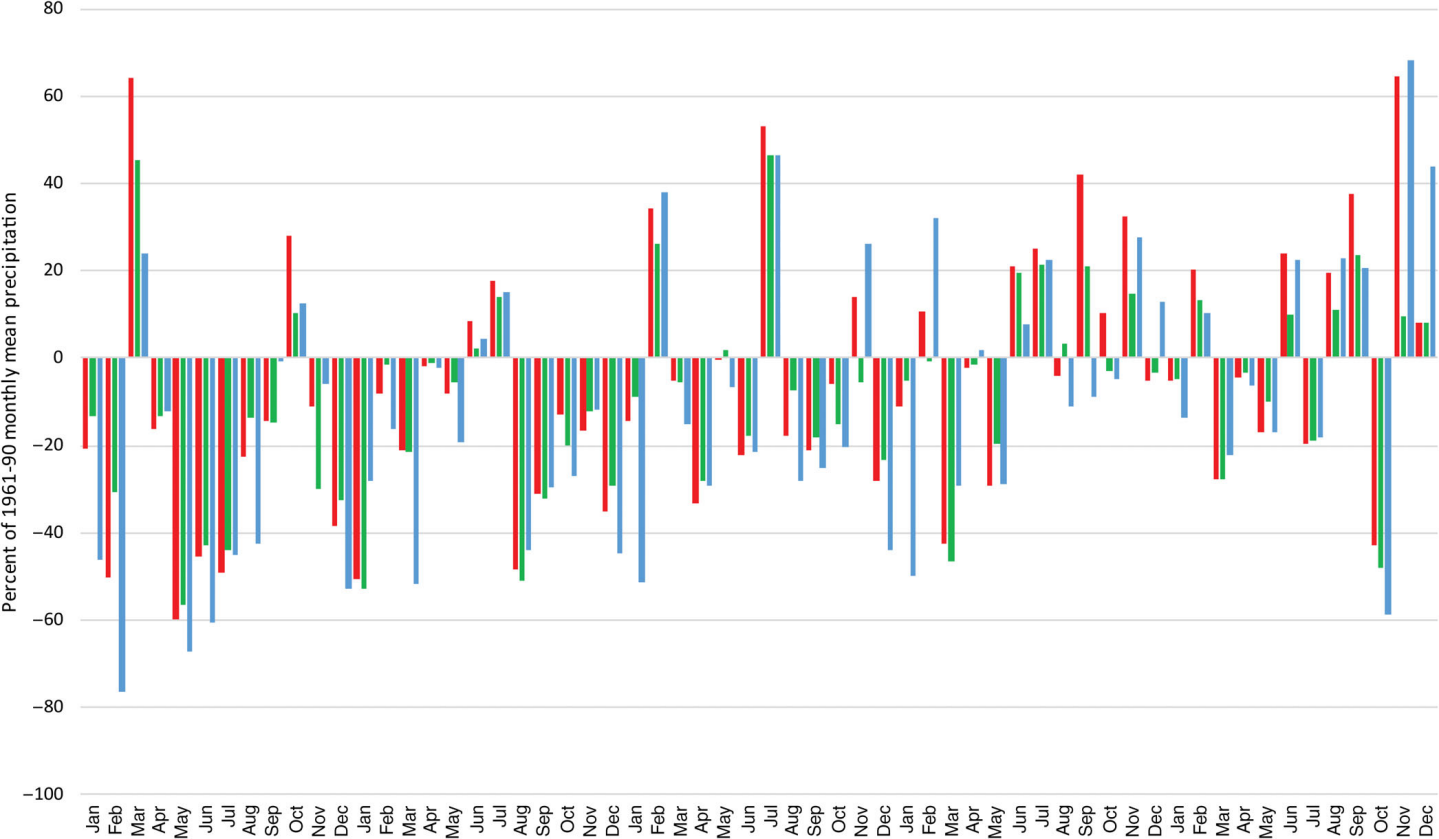
Monthly precipitation anomalies (percentage of 1961–1990 monthly mean precipitation) for January 1765 to December 1769 for the median reconstruction of EWP (red), IoI (green) and scot (blue)

From Figure 10 the 1765–1768 drought is marked by an extremely dry summer and autumn in 1765, followed by a notably dry winter in 1765–1766. While summer 1766 is not remarkable, large negative precipitation anomalies are evident from August 1766 to January 1767. While July 1767 is notably wet in each series, winter 1767–1768 is again dry, especially in Scotland. From summer 1768 onwards, monthly precipitation anomalies are more positive with the drought terminating in August and December in EWP and IoI, respectively, and by January 1769 in Scot. Evidence for social and economic impacts during summer and autumn 1765 is abundant in the newspaper records of the time from across the British‐Irish Isles. While drought continued until late 1768 (early 1769 in Scotland), the later periods of the drought are marked by dry winters and are not as clearly recorded in newspaper archives.

In Ireland, *Faulkner*'*s Dublin Journal* reports on 17 August 1765 that ‘this hath been the driest summer ever known in this kingdom, insomuch that the bed of the fine river, the Shannon, was dry at the Athlone bridge [Irish midlands]’. In England, the *Public Advertiser* reported on 4 September that ‘Letters from Newcastle of the 31st ult. contradict the accounts lately published of a great deal of rain having fallen there, and on the contrary say, that a greater drought, and want of rain, has not happened in the memory of man, than in the late season’.

Agricultural and livestock impacts were widely reported. Milk production in Ireland and England was limited owing to the lack of pasture and hay. Reporting from Waterford in southeast Ireland, the *Public Advertiser* observed on 17 August 1765 that ‘such dry weather was never known in those parts; everything burned up; the cows give no milk…’ and that ‘The mountain of Braudore is on fire, as is likewise a turf‐bog in the County of Wexford [southeast Ireland]; there is no possibility of approaching either to extinguish them’. In England the *Public Advertiser* noted on 19 July 1765 that ‘they write from Hertfordshire, Bedfordshire [Eastern and Southern England], and other adjoining counties that crops of barley, oats, beans, and peas were in general very backward, that poultry was very scarce, and the ponds all dried up for want of rain’. Similarly, on 26 July *The Derby Mercury* reported for the west of England that ‘the barley in general has suffered much for want of rain, and has an unpromising appearance’*. The Belfast Newsletter* stated on 9 August 1765 that around the London region ‘several of the livery‐stables raised the price of horse‐keeping…on account of the appearance of a scarcity of corn and hay, by reason of the great drought’. In response to the drought conditions *The Freeman*'*s Journal* on 3 August reported that ‘on account of the present long succession of dry weather, orders will speedily be issued for prayers to be read for rain, in all churches and chapels throughout England and Wales. Prayers have already been said on the above account in several churches in this metropolis’.

In addition to newspaper sources, Sinclair (1794) in his statistical account of Scotland notes that ‘In 1765, both sheep and black cattle suffered greatly from another drought, accompanied with a species of worms which destroyed the grass, by cutting its roots’. Donnelly (1978) highlights that the drought of spring and summer 1765 saw what was probably one of the most extreme droughts in Irish history. Huge losses were accrued in all aspects of agriculture, with the hay crop being so light that a fodder famine was considered inevitable, and the potato crop failed miserably throughout most of the country (Donnelly, 1978). Wilde's (1851) Census of Ireland, which also contains an extensive chronology of extreme weather events, further notes of 1765 ‘Very dry summer, with parching northeast winds. Extreme drought in Ireland in summer’ (Wilde, 1851). Dr. John Rutty, who kept a weather diary in Dublin during the period, records the following for his summary of 1765: ‘*…*The extreme drought of summer was such that some trees dropt their leaves for want of moisture, and the cattle wanted grass to supply milk and butter for the poor; and what added to the distress of these, the potatoes were scarce, and small; also there were very few apples, and the hay was scarce’.

#### 3.7.2 The 1834–1836 drought

The 1834–1836 drought was the most intense drought in the reconstructed EWP SPI‐12 series. The event is also present as an extreme drought in the reconstructed IoI series, but does not rank in the top 10 events, and is not classed as extreme in Scotland. This regional dependency is also evident in newspaper records, with most articles on the drought referring to England, and central and southern England in particular. In early summer 1834, *The Morning Chronicle* commented that the ‘long continued drought begins to be very seriously felt in the country. The grass is very short, and though the sheep find sufficient at present, the horned cattle with difficulty procure subsistence ‐ this is particularly the case in Romney Marsh [southeast England]. The cold easterly winds have brought an access of blight very prejudicial to the hops.’ By 3 June 1834 *The Morning Post* reported from Brighton [southern England] that grazing lands are parched up and cause considerable anxiety, with nights being excessively cold due to easterly winds. On 12 July 1834 *The Hampshire Telegraph* and *Sussex Chronicle* remarked that ‘In many parts of the country the excessive drought is most severely felt by cattle of all descriptions, and in some places the crops of oats have been fed off by sheep, there being no chance of their (i.e., the oats) coming to maturity.’ Reporting on the agricultural conditions of the preceding months, *The Essex County Standard* mentioned on 13 February 1835 how dry last summer was with only very scarce and localized rainfall in July, with autumn being equally dry and sunny, while many wells in the district dried up.

**Figure 11:**
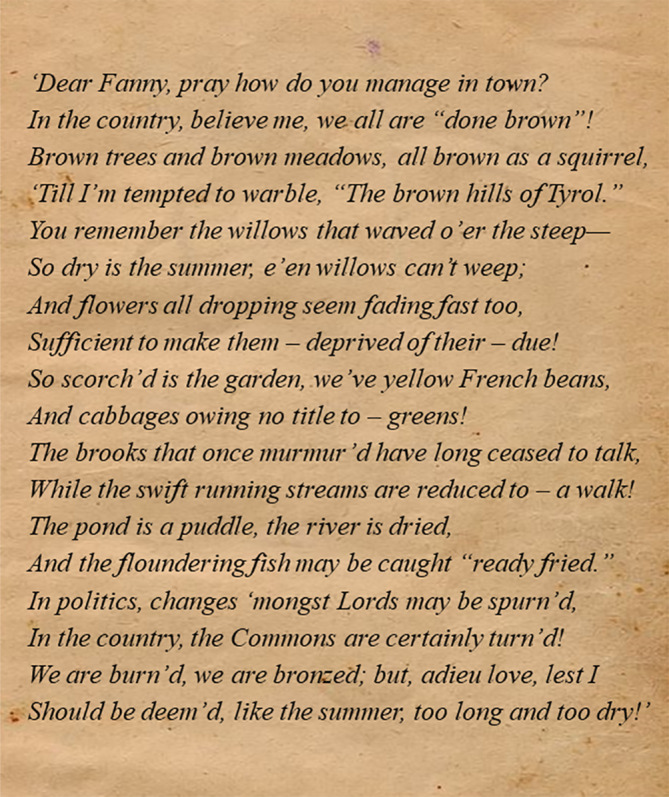
The Jeu D'esprit that appeared in Berrow's Worcester Journal on 20 August 1835 describing the impacts of drought in England

Spring and summer seem to have been equally, if not more problematic. On 5 June 1835 *The Belfast Newsletter* mentioned increased grain prices due to dry springs the last 2 years. On the 26 June *The Essex County Standard* noted that the consequences of the long drought in Romney Marsh was most disastrous and that ‘A great scarcity of water still continues to be felt in the neighbourhood of Canterbury [southeast England]. It is said the hay crop, in consequence, especially on the uplands, will be light.’ The same paper relayed on the 14 August 1835 that ‘Such has been the effect of the long‐continued drought upon the Thames, that in many places between Putney and Teddington [Greater London region] it is fordable on foot at low water.’ On 20 August, *Berrow*'*s Worcester Journal* observed that ‘The long drought has occasioned an almost unexampled deficiency in the supply of water from ponds and wells, and the keep is so short that many farmers are obliged to fodder their cattle, as in winter.’ In the very same paper, a remarkable Jeu D'esprit (light‐hearted poem; Figure 11) appeared, which was also published in several other papers around this time.

Notably, there was little coverage of drought impacts in Ireland and Scotland. However, newspapers from Ireland and England frequently mentioned the extreme nature of the 1834–1836 drought in Normandy and other parts of northwest Europe. Whistlecraft (1840) detailed the nature of the drought in England. He notes that drought conditions became established in January 1834 and continued throughout the year. In December he remarked on the continued scarcity of water and that most of the country (England) remained excessively dry—especially eastern counties (Whistlecraft, 1840, p. 138). Although there was some respite during the winter and spring of 1835, summer of that year saw the re‐establishment of drought conditions. Whistlecraft (1840), p. 153 observed for June 1835, that ‘The effects of this drought [are] daily becoming more manifest. The dryness far more general than that of last year. Reports from all parts of England bear testimony of the violence of the present drought, as being greater and more general than that of last summer, and approaching the effects of that of 1818, so memorable to our present farmers, and to that of 1800, which our elders can corroborate’. According to our median reconstruction of EWP precipitation, summer [JJA] 1800 was the driest, whereas summer 1818 ranked fifth driest. However, both tend to be shorter events and are not ranked as severe or extreme droughts when considering SPI‐12.

## 4 DISCUSSION

Reconstruction of monthly precipitation reveals an under‐catch of winter precipitation in each regional series prior to 1870. For EWP this is consistent with Murphy *et al*. (2020) and Symons (1891), who highlight that before the introduction of the Snowdon Pattern Rain Gauge in the UK in the 1860s, early winter precipitation totals are likely too low due to under‐catch of snowfall. Attribution of snow under‐catch in the observations is not so clear‐cut for IoI and Scot. For IoI much of the pre‐1830 series is based on a single weather diary or observations from sites in the UK, while Jenkinson *et al*. (1979a) adjusted the pre‐1850 IoI data using EWP. There are also no known long‐term snow observations available to evaluate model residuals. For the Scottish series the number of gauges decreases to two or less pre‐1830. Smith (1995) highlights that original estimates of early Scottish precipitation were derived by Jenkinson *et al*. (1979b), though it is unclear whether similar adjustments, as applied to the early IoI series using EWP, were applied to the Scottish series. Hence, there is a potential lack of independence in the early observations across each regional precipitation series.

For summer months, observations tend to be too high relative to our reconstructions in the pre‐1850 record, particularly for July and August. The cause is unclear, but may relate to reductions in gauge network density, the increased importance of weather diaries in the early record (IoI) and/or the spatially variable nature of summer precipitation. Adjustments by Jenkinson *et al*. (1979a, 1979b) may also be a factor, and we cannot discount non‐stationarities or in‐homogeneities in our predictors. We note, however, that regression models of July precipitation for each regional series have different predictors (EWP: LSLP, CET; IoI: DSLP, PL, CET; Scot: EDSLP), but similar deviations between observed and reconstructed precipitation.

There are several caveats and limitations to note regarding our precipitation reconstructions. The London SLP series is used to extend both the Dublin and Edinburgh SLP series, which heightens the weight attached to the London series in our results, especially for the 1765–1768 drought. However, there is strong coherence in our results for periods when the SLP series are fully independent, and documentary evidence from across the region supports our quantitative findings. The performance of regression models tends to be better for winter months; summer months have a greater amount of unexplained variance.

SPI‐12 derived from observations and median model reconstructions show close agreement for the post‐1870 period across each precipitation series, and our catalogue of major events shows good agreement with previous work (e.g., Marsh *et al*., 2007; Noone *et al*., 2017). Prior to 1870 divergence between observed and reconstructed droughts is apparent, even for some well‐established events. For instance, the 1854–1860 drought identified by Marsh *et al*. (2007) is associated with a sequence of dry winters. In terms of SPI‐12, the period is marked by two extreme droughts in 1854 and 1858, interspersed by wetter though still drier than average conditions. In our reconstructed EWP series, the 1854 event is not so extreme. Similarly, for IoI the early part of this event is not as extreme as the observations. Noone *et al*. (2017) note that the very early years of the IoI precipitation series, including the 1850s, comprises data that were extended using statistical techniques, which may account for the disparity. From our reconstructions the 1854–1860 event seems to have been most protracted in Scotland, ranking first in our reconstructed series at 53 months, and ranking in the top three events for accumulated deficit.

Large differences also emerge for 1800–1810. Noone *et al*. (2017) describe the drought of 1800–1809 in Ireland as comprising three events with brief interludes. In the UK, Cole and Marsh (2006a, 2006b) denote the long drought of 1798–1808 as three separate periods that feature among the dozen lowest 18‐month rainfall totals for EWP, but also note limited direct hydrological evidence for the event, nor substantive evidence of impacts. We find that observed SPI‐12 values for much of 1805–1810 are too low relative to our reconstructions, with the decade being less drought rich in our reconstructions. Reconstructions show an extreme drought in all three regional series for 1803–1804, with a subsequent severe drought in Scotland in 1805–1806. Thus, our findings concur with Spraggs *et al*. (2015) who find that the same decade is not as drought rich in their reconstructions for the Anglian region. We note however, that tree ring reconstructions for East Anglia show marked dryness around 1800 for spring and early summer (Cooper *et al*., 2013).

We find the 1830s to be more drought rich in our reconstructions relative to observations, with the most intense drought event in the reconstructed EWP series occurring in 1834–1836. Todd *et al*. (2013) identified this drought at Spalding but consider it locally important only, due to a lack of evidence for the event at Oxford and Kew. The extreme and widespread nature of the event is also confirmed using documentary sources, with impacts ranging from water shortages to agricultural impacts noted in English newspapers. The location and detail of sources, which range from the southeast to central and northern England, suggests that while this event likely had a larger spatial impact than asserted by Todd *et al*. (2013), the greatest impacts from the 1834–1836 drought were experienced in central, southern and southeastern England.

The most remarkable event in our reconstructions is the drought of 1765–1768, which ranks as the most extreme by most metrics across the three regional series (Table 1). This event is largely neglected by the research literature. Todd (2014) identified a major drought at Carlisle in central England from December 1765 to April 1767, but our results show that this event had a much greater spatial signature. Our findings also disagree with Cole and Marsh (2006a) who claimed that the drought in 1765 was short lived. Documentary sources confirm that a major drought occurred at this time, with significant socio‐economic impacts. Certainly, the most extreme phase of the event was in summer 1765, however, deficits seem to have persisted until 1768 in England and Ireland, and 1769 in Scotland due to a succession of dry winters. Indeed, when examined using SPI‐36 the event stands out as the most extreme multi‐year drought in all records, particularly for Scotland.

Given the emphasis placed on using historical droughts for assessing water resource resilience in the UK (Environment Agency, 2017; Environment Agency, 2018), the 1765–1768 drought provides a far sterner test for evaluating water management and investment decisions than any other drought since. While some UK water companies have turned to stochastic simulation methods to test their supply systems to events worse than those observed in historical records (e.g., Water UK, 2016), such approaches are contingent on the training data used, which is often from much shorter records. Our extended series, and the benchmark droughts within them such as 1765–1768, could give added credence to drought frequency and severity assessments based on simulation approaches. Moreover, historical assessment remains a key default stress‐testing option for a significant proportion of providers.

Finally, our results are undoubtedly sensitive to the accumulation period used to define drought. Our focus was on hydrological drought as represented by SPI‐12. Hence, intense single‐season droughts, like summer 1800—the driest summer in each of our reconstructed regional precipitations series—are not catalogued as severe or extreme droughts in our analysis. Importantly, many water supply systems (e.g., in northern and western Britain) are vulnerable to relatively short‐term, rapid onset droughts, and such events can also pose problems for other sectors (e.g., agriculture) and cause significant environmental stress. Furthermore, our work is based on regional precipitation series that represent large areas. It is plausible that the local experience of drought differs from this regional scale. Future work should: (a) investigate why observed summer precipitation in early records is apparently too wet relative to our reconstructions; (b) collate additional documentary evidence to further verify the 1765–1768 drought and (c) re‐evaluate droughts of shorter durations which may be of interest (e.g., to agriculture, by using SPI‐3) and evaluate results relative to proxy information on summer droughts from tree ring archives. In addition, the availability of new datasets for the UK (e.g., HadUK‐Grid [Hollis *et al*., 2019]) could facilitate a more spatially detailed assessment of historical droughts and could potentially be used to evaluate both observations and reconstructions of EWP and Scot precipitation. The increasing availability of historical reanalysis datasets such as 20CRv3 (Slivinski *et al*., 2019), which now extends back to 1836, also offers opportunities to further understanding of historical precipitation and drought events.

## 5 CONCLUSIONS

We reconstructed monthly precipitation for three regional precipitation series representing the British‐Irish Isles (England and Wales, Scotland and Ireland) and re‐evaluated historical droughts for the period 1748–2000. We found that observed pre‐1870 winter precipitation totals are too low across each series. While Murphy *et al*. (2020) attribute part of this bias in the EWP series to snowfall under‐catch, attribution for Scotland and Ireland is not straightforward due to possible inter‐dependencies in the data, use of weather diaries and changes in the number of contributing gauges. For summer months, observed pre‐1850 totals across each series are too high, especially in July. Following derivation of the SPI‐12 we compared severe and extreme drought events from observations and reconstructions. After 1870 we found strong agreement in the occurrence and severity of drought events. Prior to 1870, differences were identified across each regional series, even for some well‐established drought events in the 1850s and 1800s.

The most notable event in our reconstructions is the drought of 1765–1768. This ‘forgotten’ drought ranks as the most severe event, in terms of accumulated deficits across all three regional series, and the most intense event in Ireland and Scotland. Documentary evidence, predominantly from newspaper articles, confirms its existence and provides initial insight into its socio‐economic impact. Given the sensitivity of water supply systems to protracted drought events, especially in groundwater dominated systems, the drought of 1765–1768 reframes what is possible in terms of drought magnitude in the British‐Irish Isles. As such, this re‐discovered event should be a valuable benchmark against which current systems and design standards can be stress‐tested to ensure resilience to the ‘worst drought on record’ in over more than 250 years.

## Supporting information


**Appendix** **S1:** SUPPORTING INFORMATION

**Table S1** Monthly adjustment factors derived for extending Dublin (Dub) and Edinburgh (Ed) Sea level Pressure using London Sea Level pressure.
**Table S2** Selected model structures for monthly precipitation for each regional precipitation series. Also included is the median and range (in brackets) of Pearson's correlation (*r*) between observed and reconstructed precipitation from each of the 1,000 bootstrapped simulations for calibration (Cal.) and Validation (Val.) periods. The percentage of simulations with *p*‐value <.05 for model assumptions during the calibration period are also presented. These include the Durbin‐Watson (DW) test for autocorrelated residuals, the non‐constant variance (NCV) test for homoscedasticity in the residuals and the Shapiro Wilks (SW) test for normally distributed residuals.
**Table S3** Drought events identified from observed EWP SPI‐12, together with their start and end dates and associated statistics for each drought event.
**Table S4** As Table S3 but for reconstructed EWP SPI‐12.
**Table S5** As Table S3 but for observed IoI SPI‐12.
**Table S6** As Table S3 but for reconstructed IoI SPI‐12.
**Table S7** As Table S3 but for observed Scot SPI‐12.
**Table S8** As Table S3 but for reconstructed Scot SPI‐12.
