# Supplementary material for: The forgotten drought of 1765–1768: Reconstructing and re‐evaluating historical droughts in the British and Irish Isles
Source: Int J Climatol. 2020 Feb 25;40(12):5329–51. doi: 10.1002/joc.6521 (PMC7818482; doi:10.1002/joc.6521)
Supplement: Supplementary file 2 — Table S1 Monthly adjustment factors derived for extending Dublin (Dub) and Edinburgh (Ed) Sea level Pressure using London Sea Level pressure. Table S2 Selected model structures for monthly precipitation for each regional precipitation series. Also included is the median and range (in brackets) of Pearson's correlation (r) between observed and reconstructed precipitation from each of the 1,000 bootstrapped simulations for calibration (Cal.) and Validation (Val.) periods. The percentage of simulations with p‐value <.05 for model assumptions during the calibration period are also presented. These include the Durbin‐Watson (DW) test for autocorrelated residuals, the non‐constant variance (NCV) test for homoscedasticity in the residuals and the Shapiro Wilks (SW) test for normally distributed residuals. Table S3 Drought events identified from observed EWP SPI‐12, together with their start and end dates and associated statistics for each drought event. Table S4 As Table S3 but for reconstructed EWP SPI‐12. Table S5 As Table S3 but for observed IoI SPI‐12. Table S6 As Table S3 but for reconstructed IoI SPI‐12. Table S7 As Table S3 but for observed Scot SPI‐12. Table S8 As Table S3 but for reconstructed Scot SPI‐12. [file JOC-40-5329-s002.docx]

**Supplementary Information**

**The forgotten drought of 1765-1768: Reconstruction and re-evaluation of historical droughts for the British-Irish Isles.**

**Table S1 Monthly adjustment factors derived for extending Dublin (Dub) and Edinburgh (Ed) Sea level Pressure using London Sea Level pressure.**

|  | **Jan** | **Feb** | **Mar** | **Apr** | **May** | **Jun** | **Jul** | **Aug** | **Sept** | **Oct** | **Nov** | **Dec** |
| --- | --- | --- | --- | --- | --- | --- | --- | --- | --- | --- | --- | --- |
| **Ed** | 0.996 | 0.996 | 0.998 | 0.999 | 1.000 | 0.999 | 0.998 | 0.998 | 0.998 | 0.997 | 0.997 | 0.996 |
| **Dub** | 0.997 | 0.998 | 0.999 | 1.001 | 1.000 | 0.998 | 0.999 | 0.999 | 0.999 | 0.998 | 0.998 | 0.997 |

**Table S2 Selected model structures for monthly precipitation for each regional precipitation series. Also included is the median and range (in brackets) of Pearson’s correlation (r) between observed and reconstructed precipitation from each of the 1000 bootstrapped simulations for calibration (Cal.) and Validation (Val.) periods. The percentage of simulations with p-value < 0.05 for model assumptions during the calibration period are also presented. These include the Durbin-Watson (DW) test for autocorrelated residuals, the non-constant variance (NCV) test for homoscedasticity in the residuals and the Shapiro Wilks (SW) test for normally distributed residuals.**

| **EWP Month** | **Model** | **r Cal.**  **(1900-2000)** | **r Val.**  **(1870-1899)** | **DW Test** | **NCV Test** | **SW Test** |
| --- | --- | --- | --- | --- | --- | --- |
| January | jan~lslp.jan+pl.jan | 0.87 (0.77-0.94) | 0.86(0.82-0.87) | 4.4 | 3.7 | 2.8 |
| February | sqrt.feb~lslp.feb+pl.feb | 0.91 (0.83-0.95) | 0.87(0.84-0.87) | 5.8 | 5 | 2.8 |
| March | sqrt.mar~lslp.mar+pl.mar | 0.86 (0.76-0.92) | 0.83(0.80-0.83) | 5.5 | 3 | 0.7 |
| April | sqrt.apr~lslp.apr+pl.apr+cet.apr | 0.88 (0.77-0.95) | 0.91(0.85-0.92) | 5.6 | 27.6 | 8.4 |
| May | sqrt.may~lslp.may+pl.may+cet.may | 0.85 (0.66-0.92) | 0.83(0.81-0.84) | 5.5 | 0.1 | 5.1 |
| June | jun~lslp.jun+pl.jun | 0.83 (0.72-0.91) | 0.87(0.83-0.88) | 4.4 | 7.4 | 6 |
| July | jul~lslp.jul+cet.jul | 0.81 (0.66-0.91) | 0.84(0.80-0.85) | 3.8 | 3.9 | 5.7 |
| August | aug~lslp.aug | 0.90 (0.82-0.94) | 0.89(0.89-0.89) | 5.2 | 14.8 | 0.2 |
| September | sqrt.sept~lslp.sept+pl.sept+cet.sept | 0.91 (0.84-0.97) | 0.83(0.80-0.83) | 5.2 | 0.2 | 7.6 |
| October | oct~lslp.oct+cet.oct | 0.86 (0.76-0.92) | 0.86(0.81-0.87) | 5 | 7.3 | 17.3 |
| November | sqrt.nov~lslp.nov+pl.nov | 0.91 (0.85-0.94) | 0.87(0.83-0.87) | 3.9 | 4.4 | 3.1 |
| December | sqrt.dec~lslp.dec+pl.dec | 0.91 (0.82-0.95) | 0.93(0.89-0.93) | 4.7 | 2 | 0 |

| **IoI Month** | **Model** | **r Cal.**  **(1900-2000)** | **r Val.**  **(1870-1899)** | **DW Test** | **NCV Test** | **SW Test** |
| --- | --- | --- | --- | --- | --- | --- |
| January | jan~dslp.jan+pl.jan | 0.80 (0.64-0.89) | 0.82 (0.74-0.83) | 6.3 | 28.5 | 0.5 |
| February | feb~dslp.feb+pl.feb | 0.85 (0.70-0.93) | 0.82 (0.78-0.82) | 5.1 | 7.8 | 14.5 |
| March | sqrt.mar~dslp.mar+pl.mar | 0.76 (0.65-0.87) | 0.72 (0.70-0.72) | 5.3 | 7.4 | 3.9 |
| April | sqrt.apr~dslp.apr+pl.apr | 0.77 (0.56-0.89) | 0.76 (072-0.76) | 5.6 | 3 | 40 |
| May | sqrt.may~dslp.may+pl.may | 0.81 (0.66-0.90) | 0.78 (0.72-0.80) | 4 | 0 | 7.2 |
| June | sqrt.jun~dslp.jun+pl.jun | 0.77 (0.60-0.86) | 0.76 (0.72-0.76) | 4.4 | 0 | 17.2 |
| July | jul~dslp.jul+pl.jul+cet.jul | 0.78 (0.61-0.90) | 0.68 (0.63-0.68) | 6.2 | 2.1 | 1.4 |
| August | aug~dslp.aug+pl.aug | 0.76 (0.53-0.89) | 0.77 (0.64-0.80) | 5.5 | 1 | 8.4 |
| September | sept~dslp.sept+pl.sept | 0.83 (0.71-0.91) | 0.74 (0.70-0.75) | 5.3 | 0.5 | 9.5 |
| October | oct~dslp.oct+pl.oct+cet.oct | 0.78 (0.65-0.88) | 0.75 (0.68-0.77) | 4.3 | 3.9 | 1.5 |
| November | nov~dslp.nov+pl.nov+cet.nov | 0.79 (0.64-0.89) | 0.77 (0.70-0.78) | 5 | 0.3 | 1.7 |
| December | sqrt.dec~dslp.dec+pl.dec | 0.80 (0.60-0.90) | 0.76 (0.63-0.78) | 3.7 | 0.2 | 2.8 |

| **Scot Month** | **Model** | **r Cal.**  **(1900-2000)** | **r Val.**  **(1870-1899)** | **DW Test** | **NCV Test** | **SW Test** |
| --- | --- | --- | --- | --- | --- | --- |
| January | jan~eslp.jan+pl.jan+cet.jan | 0.82 (0.66-0.89) | 0.88 (0.79-0.92) | 3.2 | 6.9 | 5.9 |
| February | sqrt. feb~eslp.feb+pl.feb | 0.89 (0.81-0.94) | 0.80 (0.74-0.83) | 5.2 | 11.9 | 6 |
| March | sqrt.mar~pl.mar | 0.79 (0.65-0.88) | 0.74 (0.74-0.74) | 4.6 | 6 | 2.9 |
| April | sqrt.apr~eslp.apr+pl.apr | 0.80 (0.63-0.90) | 0.83 (0.76-0.84) | 5.2 | 8.9 | 1.8 |
| May | sqrt.may~eslp.may+pl.may | 0.77 (0.62-0.88) | 0.79 (0.72-0.80) | 4.9 | 3 | 2.6 |
| June | sqrt.jun~eslp.jun+pl.jun | 0.83 (0.70-0.90) | 0.76 (0.74-0.76) | 4.4 | 4 | 7 |
| July | sqrt.jul~eslp.jul | 0.72 (0.54-0.84) | 0.82 (0.82-0.82) | 4.4 | 4.4 | 8.7 |
| August | aug~eslp.aug+pl.aug | 0.87 (0.75-0.92) | 0.83 (0.81-0.84) | 5 | 8.9 | 17.7 |
| September | sqrt.sept~eslp.sept+pl.sept | 0.84 (0.71-0.92) | 0.86 (0.81-0.87) | 4.9 | 1.9 | 4 |
| October | oct~eslp.oct+cet.oct | 0.82 (0.71-0.89) | 0.74 (0.65-0.74) | 3.7 | 1.4 | 5 |
| November | nov~pl.nov+eslp.nov | 0.84 (0.75-0.91) | 0.68 (0.63-0.69) | 5.9 | 4.5 | 7 |
| December | dec~eslp.dec+pl.dec | 0.81 (0.66-0.91) | 0.69 (0.55-0.77) | 4.5 | 6.5 | 1.9 |

**Table S3 Drought events identified from observed EWP SPI-12, together with their start and end dates and associated statistics for each drought event.**

| **ID** | **Start Year/Month** | **End Year/Month** | **Duration (months)** | **Mean SPI-12** | **Accumulated SPI-12** | **Min SPI-12** |
| --- | --- | --- | --- | --- | --- | --- |
| 1 | 176612 | 176702 | 2 | -0.66 | -1.32 | -1.11 |
| 2 | 177112 | 177204 | 4 | -0.69 | -2.78 | -1.24 |
| 3 | 177702 | 177707 | 5 | -0.77 | -3.87 | -1.34 |
| 4 | 177709 | 177909 | 24 | -0.77 | -18.44 | -1.58 |
| 5 | 178012 | 178205 | 17 | -1.74 | -29.66 | -2.91 |
| 6 | 178401 | 178707 | 42 | -1.20 | -50.57 | -3.86 |
| 7 | 178810 | 178910 | 12 | -1.43 | -17.17 | -3.26 |
| 8 | 179010 | 179102 | 4 | -0.62 | -2.48 | -1.08 |
| 9 | 179310 | 179503 | 17 | -1.01 | -17.12 | -1.60 |
| 10 | 179702 | 179708 | 6 | -0.78 | -4.68 | -1.39 |
| 11 | 180009 | 180102 | 5 | -0.74 | -3.70 | -1.21 |
| 12 | 180209 | 180408 | 23 | -1.23 | -28.22 | -2.73 |
| 13 | 180510 | 180902 | 40 | -1.17 | -46.99 | -1.96 |
| 14 | 181002 | 181011 | 9 | -1.89 | -8.01 | -1.57 |
| 15 | 181402 | 181607 | 29 | -0.88 | -25.66 | -1.76 |
| 16 | 182004 | 182111 | 19 | -0.95 | -18.04 | -1.58 |
| 17 | 182606 | 182712 | 18 | -1.12 | -20.08 | -2.14 |
| 18 | 183501 | 183601 | 12 | -0.88 | -10.56 | -1.84 |
| 19 | 183801 | 183901 | 12 | -0.74 | -8.91 | -1.48 |
| 20 | 184012 | 184109 | 9 | -0.77 | -6.89 | -1.34 |
| 21 | 184406 | 184603 | 21 | -1.36 | -28.48 | -2.47 |
| 22 | 184708 | 184803 | 7 | -0.97 | -6.82 | -1.52 |
| 23 | 185012 | 185103 | 3 | -0.81 | -2.42 | -1.15 |
| 24 | 185112 | 185209 | 9 | -1.22 | -10.94 | -1.74 |
| 25 | 185404 | 185609 | 29 | -1.59 | -46.22 | -3.06 |
| 26 | 185801 | 185911 | 22 | -1.21 | -26.51 | -2.15 |
| 27 | 186401 | 186601 | 24 | -1.04 | -25.03 | -2.19 |
| 28 | 186806 | 186902 | 8 | -1.13 | -9.01 | -2.04 |
| 29 | 187005 | 187109 | 16 | -1.20 | -19.13 | -1.94 |
| 30 | 187401 | 187506 | 17 | -0.96 | -16.35 | -1.79 |
| 31 | 088008 | 188011 | 3 | -1.10 | -3.31 | -1.63 |
| 32 | 088411 | 188510 | 11 | -1.18 | -12.98 | -1.62 |
| 33 | 188707 | 188905 | 22 | -1.11 | -24.36 | -2.73 |
| 34 | 189010 | 189110 | 12 | -1.15 | -13.82 | -1.49 |
| 35 | 189306 | 189408 | 14 | -1.02 | -14.31 | -1.84 |
| 36 | 189601 | 189702 | 13 | -0.88 | -11.39 | -1.59 |
| 37 | 189803 | 190008 | 29 | -0.71 | -20.51 | -2.06 |
| 38 | 190111 | 190307 | 20 | -0.92 | -18.36 | -1.60 |
| 39 | 190412 | 190612 | 24 | -1.05 | -25.17 | -2.19 |
| 40 | 190702 | 190802 | 12 | -0.42 | -5.10 | -1.14 |
| 41 | 190812 | 190912 | 12 | -0.96 | -11.46 | -1.31 |
| 42 | 191108 | 191203 | 7 | -0.69 | -4.83 | -1.22 |
| 43 | 191701 | 191412 | 11 | -0.77 | -8.45 | -1.14 |
| 44 | 192105 | 192209 | 16 | -1.82 | -26.19 | -3.05 |
| 45 | 192903 | 193001 | 10 | -1.15 | -11.47 | -1.79 |
| 46 | 193310 | 193506 | 20 | -1.60 | -32.05 | -2.57 |
| 47 | 193803 | 193901 | 10 | -1.30 | -13.04 | -1.91 |
| 48 | 194203 | 194502 | 35 | -0.86 | -30.10 | -1.64 |
| 49 | 194803 | 194810 | 7 | -0.92 | -6.47 | -1.35 |
| 50 | 194906 | 195007 | 13 | -0.98 | -12.80 | -2.30 |
| 51 | 195312 | 195410 | 10 | -0.73 | -7.34 | -1.63 |
| 52 | 195511 | 195703 | 16 | -1.01 | -16.21 | -2.21 |
| 53 | 195909 | 196002 | 5 | -1.36 | -6.80 | -2.01 |
| 54 | 196212 | 196311 | 11 | -1.03 | -11.29 | -1.73 |
| 55 | 196408 | 196509 | 13 | -1.32 | -17.19 | -2.48 |
| 56 | 197208 | 197410 | 26 | -1.05 | -27.38 | -1.79 |
| 57 | 197511 | 197702 | 15 | -2.09 | -31.37 | -3.35 |
| 58 | 198407 | 198411 | 4 | -0.76 | -3.03 | -1.03 |
| 59 | 198907 | 199001 | 6 | -1.30 | -7.77 | -1.67 |
| 60 | 199102 | 199211 | 21 | -0.99 | -20.73 | -1.70 |
| 61 | 199601 | 199801 | 24 | -1.39 | -33.48 | -2.06 |

**Table S4 As Table S3 but for reconstructed EWP SPI-12.**

| **ID** | **Start Year/Month** | **End Year/Month** | **Duration (months)** | **Mean SPI-12** | **Accumulated SPI-12** | **Min SPI-12** |
| --- | --- | --- | --- | --- | --- | --- |
| 1 | 175210 | 175406 | 20 | -0.80 | -15.91 | -1.51 |
| 2 | 176301 | 176306 | 5 | -0.81 | -4.06 | -1.29 |
| 3 | 176506 | 176810 | 40 | -1.52 | -60.97 | -3.19 |
| 4 | 177808 | 177811 | 3 | -0.84 | -2.52 | -1.08 |
| 5 | 178103 | 178208 | 17 | -1.47 | -24.93 | -2.69 |
| 6 | 178308 | 178407 | 11 | -1.14 | -12.58 | -1.77 |
| 7 | 178506 | 178605 | 11 | -1.17 | -12.85 | -2.22 |
| 8 | 178701 | 178707 | 6 | -0.56 | -3.33 | -1.01 |
| 9 | 178812 | 178905 | 5 | -0.64 | -3.22 | -1.13 |
| 10 | 179010 | 179106 | 8 | -0.69 | -5.53 | -1.66 |
| 11 | 179310 | 179411 | 13 | -0.81 | -10.55 | -1.12 |
| 12 | 179701 | 179710 | 9 | -1.18 | -10.64 | -1.67 |
| 13 | 180307 | 180403 | 8 | -1.20 | -9.60 | -2.31 |
| 14 | 180511 | 180601 | 2 | -0.71 | -1.43 | -1.01 |
| 15 | 181301 | 181505 | 18 | -0.94 | -26.35 | -1.63 |
| 16 | 182212 | 182302 | 2 | -0.86 | -1.72 | -1.08 |
| 17 | 182612 | 182802 | 14 | -0.88 | -12.32 | -1.81 |
| 18 | 183004 | 183012 | 8 | -0.52 | -4.13 | -1.00 |
| 19 | 183210 | 183311 | 13 | -1.10 | -14.33 | -2.15 |
| 20 | 183411 | 183603 | 16 | -1.78 | -28.41 | -3.29 |
| 21 | 183712 | 183901 | 13 | -0.70 | -9.15 | -1.44 |
| 22 | 184212 | 184305 | 5 | -0.69 | -3.45 | -1.43 |
| 23 | 185203 | 185209 | 6 | -1.09 | -6.52 | -1.65 |
| 24 | 158409 | 185609 | 24 | -1.04 | -24.98 | -1.93 |
| 25 | 185712 | 185910 | 22 | -1.27 | -28.03 | -2.10 |
| 26 | 186805 | 186902 | 9 | -1.01 | -9.11 | -1.74 |
| 27 | 187005 | 187107 | 14 | -0.82 | -11.42 | -1.39 |
| 28 | 187601 | 187603 | 2 | -1.00 | -2.01 | -1.29 |
| 29 | 188006 | 188101 | 7 | -1.26 | -8.82 | -1.99 |
| 30 | 188710 | 188806 | 8 | -1.10 | -8.82 | -1.70 |
| 31 | 189605 | 189610 | 5 | -0.90 | -4.51 | -1.41 |
| 32 | 189808 | 189901 | 5 | -0.84 | -4.18 | -1.56 |
| 33 | 190501 | 190602 | 13 | -0.98 | -12.70 | -1.83 |
| 34 | 190609 | 190710 | 13 | -0.97 | -12.59 | -1.85 |
| 35 | 190812 | 190910 | 10 | -0.81 | -8.10 | -1.30 |
| 36 | 191107 | 191202 | 7 | -0.91 | -6.37 | -1.35 |
| 37 | 192104 | 192209 | 17 | -1.41 | -24.01 | -2.42 |
| 38 | 192904 | 193001 | 9 | -1.31 | -11.81 | -1.61 |
| 39 | 193208 | 193210 | 2 | -0.89 | -1.78 | -1.15 |
| 40 | 193310 | 193412 | 14 | -1.23 | -17.25 | -1.82 |
| 41 | 193803 | 193903 | 12 | -0.90 | -10.80 | -1.67 |
| 42 | 194001 | 194102 | 13 | -0.91 | -11.80 | -1.39 |
| 43 | 194202 | 194302 | 12 | -1.32 | -15.86 | -2.01 |
| 44 | 194402 | 194505 | 15 | -0.86 | -12.86 | -1.38 |
| 45 | 194511 | 194611 | 12 | -0.63 | -7.58 | -1.09 |
| 46 | 194803 | 194810 | 7 | -0.93 | -6.52 | -1.28 |
| 47 | 194901 | 195007 | 18 | -1.43 | -25.70 | -2.80 |
| 48 | 195310 | 195408 | 10 | -0.93 | -9.29 | -1.56 |
| 49 | 195602 | 195703 | 13 | -0.82 | -10.68 | -1.57 |
| 50 | 195908 | 196002 | 6 | -0.99 | -5.91 | -1.92 |
| 51 | 196301 | 196311 | 10 | -0.85 | -8.48 | -1.82 |
| 52 | 196411 | 196509 | 10 | -0.76 | -7.56 | -1.58 |
| 53 | 197111 | 197409 | 34 | -1.40 | -47.59 | -2.67 |
| 54 | 197511 | 197703 | 16 | -2.05 | -32.87 | -3.09 |
| 55 | 198405 | 198411 | 6 | -0.87 | -5.23 | -1.20 |
| 56 | 198705 | 198710 | 5 | -0.80 | -4.02 | -1.22 |
| 57 | 198907 | 199010 | 15 | -0.91 | -13.58 | -1.94 |
| 58 | 199102 | 199301 | 23 | -1.36 | -31.19 | -2.37 |
| 59 | 199602 | 199801 | 23 | -1.21 | -27.77 | -1.74 |

**Table S5 As Table S3 but for observed IoI SPI-12.**

| **ID** | **Start Year/Month** | **End Year/Month** | **Duration (months)** | **Mean SPI-12** | **Accumulated SPI-12** | **Min SPI-12** |
| --- | --- | --- | --- | --- | --- | --- |
| 1 | 174812 | 175105 | 29 | -1.43 | -41.61 | -2.62 |
| 2 | 175501 | 175509 | 8 | -1.07 | -8.53 | -1.78 |
| 3 | 175710 | 175802 | 4 | -0.72 | -2.89 | -1.22 |
| 4 | 175907 | 176012 | 17 | -1.35 | -22.90 | -1.97 |
| 5 | 176206 | 176307 | 13 | -1.49 | -19.32 | -2.22 |
| 6 | 176509 | 176801 | 28 | -1.28 | -35.93 | -3.27 |
| 7 | 177009 | 177011 | 2 | -1.38 | -2.75 | -1.56 |
| 8 | 177111 | 177206 | 7 | -1.06 | -7.45 | -1.85 |
| 9 | 177501 | 177510 | 9 | -0.74 | -6.64 | -1.40 |
| 10 | 177709 | 177807 | 10 | -0.52 | -5.19 | -1.42 |
| 11 | 178112 | 178205 | 17 | -1.34 | -22.85 | -2.34 |
| 12 | 178410 | 178711 | 37 | -1.57 | -57.93 | -4.84 |
| 13 | 178810 | 178911 | 13 | -2.39 | -31.08 | -4.12 |
| 14 | 179310 | 179411 | 13 | -1.08 | -14.04 | -1.75 |
| 15 | 179611 | 179801 | 14 | -1.87 | -26.25 | -3.06 |
| 16 | 179901 | 179905 | 4 | -0.69 | -2.75 | -1.32 |
| 17 | 180008 | 180208 | 24 | -0.95 | -22.70 | -1.59 |
| 18 | 180307 | 180410 | 15 | -1.38 | -20.74 | -2.80 |
| 19 | 180510 | 180611 | 13 | -1.47 | -19.06 | -1.87 |
| 20 | 181008 | 181104 | 8 | -0.86 | -6.90 | -1.74 |
| 21 | 181401 | 181502 | 13 | -1.54 | -20.05 | -2.38 |
| 22 | 181603 | 181607 | 4 | -0.83 | -3.32 | -1.07 |
| 23 | 182002 | 182112 | 22 | -1.06 | -23.23 | -1.88 |
| 24 | 182405 | 182506 | 13 | -1.19 | -15.42 | -2.82 |
| 25 | 182611 | 182801 | 14 | -1.30 | -18.23 | -3.08 |
| 26 | 182912 | 183112 | 24 | -0.91 | -21.73 | -1.73 |
| 27 | 183211 | 183311 | 12 | -0.79 | -9.49 | -1.41 |
| 28 | 183501 | 183511 | 10 | -0.89 | -8.90 | -1.61 |
| 29 | 183711 | 183909 | 22 | -0.56 | -12.23 | -1.22 |
| 30 | 184010 | 184202 | 16 | -1.27 | -20.25 | -2.26 |
| 31 | 184210 | 184603 | 41 | -0.90 | -37.00 | -2.08 |
| 32 | 184708 | 184803 | 7 | -1.07 | -7.51 | -1.90 |
| 33 | 184908 | 185002 | 6 | -0.37 | -2.22 | -1.08 |
| 34 | 185201 | 185211 | 10 | -0.70 | -7.03 | -1.18 |
| 35 | 185404 | 186005 | 73 | -1.38 | -100.84 | -2.98 |
| 36 | 186410 | 186508 | 10 | -0.97 | -9.75 | -1.69 |
| 37 | 186807 | 186812 | 5 | -1.05 | -5.27 | -1.45 |
| 38 | 187004 | 187107 | 15 | -1.10 | -16.52 | -2.09 |
| 39 | 187401 | 187501 | 12 | -1.19 | -14.27 | -1.93 |
| 40 | 188008 | 188106 | 10 | -0.64 | -6.44 | -1.83 |
| 41 | 188503 | 188510 | 7 | -0.84 | -5.88 | -1.31 |
| 42 | 188707 | 188902 | 19 | -1.59 | -30.30 | -3.23 |
| 43 | 188907 | 188910 | 3 | -0.75 | -2.25 | -1.26 |
| 44 | 189010 | 189202 | 16 | -1.14 | -18.18 | -2.03 |
| 45 | 189308 | 189411 | 15 | -1.18 | -17.71 | -2.57 |
| 46 | 189505 | 189609 | 16 | -0.76 | -12.10 | -1.41 |
| 47 | 190210 | 190302 | 4 | -0.67 | -2.67 | -1.09 |
| 48 | 190502 | 190802 | 36 | -1.19 | -42.82 | -2.13 |
| 49 | 190908 | 191008 | 12 | -0.78 | -9.37 | -1.23 |
| 50 | 191106 | 191202 | 8 | -1.31 | -10.51 | -2.35 |
| 51 | 191406 | 191408 | 2 | -0.85 | -1.71 | -1.10 |
| 52 | 191808 | 191809 | 1 | -1.04 | -1.04 | -1.04 |
| 53 | 191910 | 192007 | 9 | -1.09 | -9.81 | -1.69 |
| 54 | 192106 | 192310 | 28 | -1.03 | -28.92 | -1.86 |
| 55 | 192702 | 192709 | 7 | -0.70 | -4.88 | -1.13 |
| 56 | 193211 | 193412 | 25 | -1.52 | -37.99 | -3.31 |
| 57 | 193802 | 193811 | 9 | -1.20 | -10.82 | -2.13 |
| 58 | 194112 | 194209 | 9 | -0.88 | -7.96 | -1.35 |
| 59 | 194211 | 194311 | 12 | -0.49 | -5.82 | -1.11 |
| 60 | 194402 | 194502 | 12 | -1.17 | -14.05 | -1.88 |
| 61 | 194912 | 195007 | 7 | -0.60 | -4.22 | -1.10 |
| 62 | 195209 | 195406 | 21 | -1.46 | -30.76 | -2.65 |
| 63 | 195511 | 195702 | 15 | -1.03 | -15.38 | -2.45 |
| 64 | 195908 | 196002 | 6 | -1.38 | -8.26 | -2.56 |
| 65 | 196301 | 196311 | 10 | -0.92 | -9.19 | -1.54 |
| 66 | 196411 | 196501 | 2 | -0.71 | -1.42 | -1.10 |
| 67 | 196911 | 197011 | 12 | -1.17 | -14.08 | -1.78 |
| 68 | 197102 | 197402 | 36 | -1.16 | -41.81 | -2.42 |
| 69 | 197506 | 197702 | 20 | -1.60 | -32.03 | -2.54 |
| 70 | 198501 | 198506 | 5 | -0.81 | -4.06 | -1.25 |
| 71 | 198909 | 199002 | 5 | -0.87 | -4.36 | -1.15 |
| 72 | 199102 | 199104 | 2 | -0.78 | -1.57 | -1.10 |
| 73 | 199202 | 199209 | 7 | -0.98 | -6.84 | -1.29 |
| 74 | 199703 | 199709 | 6 | -0.88 | -5.28 | -1.49 |

**Table S6 As Table S3 but for reconstructed IoI SPI-12.**

| **ID** | **Start Year/Month** | **End Year/Month** | **Duration (months)** | **Mean SPI-12** | **Accumulated SPI-12** | **Min SPI-12** |
| --- | --- | --- | --- | --- | --- | --- |
| 1 | 175009 | 175103 | 6 | -0.65 | -3.90 | -1.06 |
| 2 | 175307 | 175504 | 21 | -0.60 | -12.54 | -1.31 |
| 3 | 176506 | 176812 | 42 | -1.36 | -57.00 | -3.14 |
| 4 | 177903 | 178001 | 10 | -0.66 | -6.55 | -1.07 |
| 5 | 178103 | 178205 | 14 | -1.25 | -17.43 | -2.09 |
| 6 | 178308 | 178605 | 33 | -0.83 | -24.47 | -2.17 |
| 7 | 178701 | 178707 | 6 | -0.63 | -3.76 | -1.06 |
| 8 | 178812 | 178906 | 6 | -0.61 | -3.65 | -1.05 |
| 9 | 179010 | 179103 | 5 | -0.85 | -4.24 | -1.39 |
| 10 | 179310 | 179410 | 12 | -0.63 | -7.53 | -1.06 |
| 11 | 179702 | 179712 | 10 | -0.90 | -8.97 | -1.48 |
| 12 | 180309 | 180407 | 10 | -0.77 | -7.71 | -1.80 |
| 13 | 180511 | 180603 | 4 | -0.51 | -2.03 | -1.12 |
| 14 | 181301 | 181506 | 29 | -1.14 | -33.17 | -1.79 |
| 15 | 182212 | 182302 | 2 | -0.81 | -1.61 | -1.06 |
| 16 | 182612 | 182706 | 6 | -0.81 | -4.86 | -1.28 |
| 17 | 183212 | 183312 | 12 | -0.69 | -8.28 | -1.60 |
| 18 | 183412 | 183603 | 15 | -1.24 | -18.64 | -2.20 |
| 19 | 183710 | 183909 | 23 | -0.85 | -19.48 | -1.90 |
| 20 | 184101 | 184104 | 3 | -0.68 | -2.03 | -1.02 |
| 21 | 184212 | 184305 | 5 | -0.55 | -2.75 | -1.13 |
| 22 | 184405 | 184512 | 19 | -0.98 | -18.58 | -1.74 |
| 23 | 185001 | 185103 | 14 | -0.87 | -12.13 | -1.27 |
| 24 | 185111 | 185211 | 12 | -1.64 | -19.65 | -2.93 |
| 25 | 185409 | 185609 | 24 | -0.81 | -19.54 | -1.69 |
| 26 | 185708 | 185910 | 26 | -1.27 | -33.12 | -2.25 |
| 27 | 186409 | 186606 | 21 | -1.09 | -22.98 | -1.86 |
| 28 | 186807 | 186812 | 5 | -1.03 | -5.17 | -1.21 |
| 29 | 187002 | 187108 | 18 | -1.29 | -23.26 | -2.11 |
| 30 | 187407 | 187604 | 21 | -0.75 | -15.72 | -1.76 |
| 31 | 188006 | 188112 | 18 | -0.94 | -16.87 | -2.24 |
| 32 | 188707 | 188808 | 13 | -1.56 | -20.34 | -2.59 |
| 33 | 189102 | 189110 | 8 | -1.14 | -9.13 | -1.42 |
| 34 | 189605 | 189704 | 11 | -1.08 | -11.87 | -1.97 |
| 35 | 189808 | 189901 | 5 | -0.74 | -3.71 | -1.36 |
| 36 | 190004 | 190012 | 8 | -0.59 | -4.75 | -1.24 |
| 37 | 190502 | 190710 | 32 | -0.92 | -29.37 | -1.75 |
| 38 | 191107 | 191201 | 6 | -0.84 | -5.06 | -1.45 |
| 39 | 191712 | 191812 | 12 | -0.61 | -7.30 | -1.21 |
| 40 | 191910 | 192007 | 9 | -0.92 | -8.24 | -1.31 |
| 41 | 192106 | 192209 | 15 | -1.31 | -19.65 | -2.06 |
| 42 | 192903 | 193001 | 10 | -1.36 | -13.60 | -1.73 |
| 43 | 193206 | 193303 | 9 | -0.85 | -7.63 | -1.77 |
| 44 | 193310 | 193412 | 14 | -1.43 | -19.97 | -2.03 |
| 45 | 193803 | 193902 | 11 | -1.30 | -14.26 | -2.10 |
| 46 | 193912 | 194108 | 20 | -1.21 | -24.30 | -2.08 |
| 47 | 194110 | 194302 | 16 | -1.29 | -20.70 | -2.05 |
| 48 | 194402 | 194609 | 31 | -0.73 | -22.71 | -1.75 |
| 49 | 194807 | 194810 | 3 | -0.54 | -1.63 | -1.18 |
| 50 | 194906 | 195007 | 13 | -1.08 | -14.03 | -2.20 |
| 51 | 195212 | 195410 | 22 | -1.37 | -30.12 | -2.38 |
| 52 | 195510 | 195801 | 27 | -1.08 | -29.23 | -2.49 |
| 53 | 195908 | 196002 | 6 | -1.28 | -7.68 | -2.28 |
| 54 | 196301 | 196308 | 7 | -0.77 | -5.38 | -1.70 |
| 55 | 196411 | 196509 | 10 | -0.56 | -5.58 | -1.09 |
| 56 | 197111 | 197409 | 34 | -1.14 | -38.92 | -2.61 |
| 57 | 197512 | 197702 | 14 | -1.68 | -23.50 | -2.51 |
| 58 | 198705 | 198710 | 5 | -0.89 | -4.43 | -1.22 |
| 59 | 198908 | 199001 | 5 | -1.14 | -5.72 | -1.51 |
| 60 | 199112 | 199301 | 13 | -1.15 | -14.89 | -1.84 |
| 61 | 199311 | 199402 | 3 | -0.85 | -2.55 | -1.36 |
| 62 | 199603 | 199712 | 21 | -0.85 | -17.93 | -1.48 |

**Table S7 As Table S3 but for observed Scot SPI-12.**

| **ID** | **Start Year/Month** | **End Year/Month** | **Duration (months)** | **Mean SPI-12** | **Accumulated SPI-12** | **Min SPI-12** |
| --- | --- | --- | --- | --- | --- | --- |
| 1 | 175712 | 175812 | 12 | -1.35 | -16.16 | -2.35 |
| 2 | 176006 | 176012 | 6 | -1.19 | -7.16 | -1.94 |
| 3 | 176211 | 176308 | 9 | -2.18 | -19.58 | -3.09 |
| 4 | 176501 | 176702 | 25 | -2.03 | -50.74 | -4.92 |
| 5 | 176706 | 176711 | 5 | -0.44 | -2.20 | -1.27 |
| 6 | 177009 | 177012 | 3 | -0.74 | -2.22 | -1.05 |
| 7 | 177106 | 177110 | 4 | -1.34 | -5.35 | -1.73 |
| 8 | 177112 | 177206 | 6 | -0.76 | -4.54 | -1.13 |
| 9 | 177610 | 177811 | 25 | -1.00 | -15.08 | -2.14 |
| 10 | 178012 | 178203 | 15 | -2.18 | -32.74 | -3.55 |
| 11 | 178305 | 178712 | 55 | -1.65 | -90.86 | -5.38 |
| 12 | 178808 | 178911 | 15 | -2.21 | -33.15 | -3.58 |
| 13 | 179403 | 179410 | 7 | -0.72 | -5.05 | -1.19 |
| 14 | 179611 | 179712 | 13 | -2.42 | -31.51 | -3.71 |
| 15 | 179907 | 179909 | 2 | -0.60 | -1.20 | -1.13 |
| 16 | 180008 | 180709 | 85 | -1.76 | -149.24 | -3.19 |
| 17 | 181008 | 181102 | 6 | -1.00 | -6.00 | -1.70 |
| 18 | 181308 | 181509 | 25 | -2.01 | -50.18 | -4.09 |
| 19 | 181603 | 181706 | 15 | -0.62 | -9.37 | -1.22 |
| 20 | 181808 | 181912 | 16 | -0.64 | -10.19 | -1.04 |
| 21 | 182002 | 182202 | 24 | -1.07 | -25.79 | -2.14 |
| 22 | 182408 | 182712 | 40 | -1.19 | -47.58 | -2.61 |
| 23 | 182905 | 182909 | 4 | -0.83 | -3.32 | -1.11 |
| 24 | 183109 | 183201 | 4 | -0.45 | -1.79 | -1.41 |
| 25 | 183501 | 183511 | 10 | -0.64 | -6.42 | -1.45 |
| 26 | 183801 | 183901 | 12 | -0.71 | -8.54 | -1.73 |
| 27 | 183908 | 184001 | 5 | -0.57 | -2.86 | -1.44 |
| 28 | 184212 | 184311 | 11 | -0.91 | -10.01 | -1.93 |
| 29 | 184410 | 184512 | 14 | -1.64 | -22.94 | -2.31 |
| 30 | 184707 | 184802 | 7 | -1.43 | -9.99 | -1.91 |
| 31 | 185001 | 185101 | 12 | -0.86 | -10.29 | -1.43 |
| 32 | 185112 | 185212 | 12 | -1.39 | -16.64 | -2.04 |
| 33 | 185312 | 185903 | 63 | -1.59 | -100.19 | -2.93 |
| 34 | 186004 | 186108 | 16 | -0.58 | -9.29 | -1.19 |
| 35 | 186509 | 186609 | 12 | -0.92 | -11.08 | -1.46 |
| 36 | 186908 | 187203 | 31 | -1.33 | -41.23 | -2.41 |
| 37 | 187601 | 187603 | 2 | -0.91 | -1.81 | -1.27 |
| 38 | 187812 | 187908 | 8 | -1.12 | -8.94 | -1.66 |
| 39 | 187912 | 188204 | 28 | -1.51 | -42.18 | -3.16 |
| 40 | 188507 | 189011 | 64 | -1.48 | -94.80 | -2.74 |
| 41 | 189106 | 189202 | 8 | -0.98 | -7.80 | -1.72 |
| 42 | 189301 | 189402 | 13 | -1.01 | -13.19 | -1.53 |
| 43 | 189502 | 189811 | 45 | -0.90 | -40.36 | -2.33 |
| 44 | 190202 | 190303 | 13 | -1.21 | -15.72 | -1.74 |
| 45 | 190411 | 190607 | 20 | -0.95 | -19.01 | -1.74 |
| 46 | 190903 | 191002 | 11 | -0.63 | -6.91 | -1.38 |
| 47 | 191108 | 191203 | 7 | -0.99 | -6.91 | -1.93 |
| 48 | 191312 | 191502 | 14 | -1.02 | -14.28 | -1.82 |
| 49 | 191707 | 191802 | 7 | -0.61 | -4.27 | -1.28 |
| 50 | 191909 | 192002 | 5 | -1.19 | -5.97 | -1.66 |
| 51 | 192105 | 192207 | 14 | -0.77 | -10.82 | -1.30 |
| 52 | 192301 | 192309 | 8 | -0.92 | -7.37 | -1.15 |
| 53 | 192605 | 192610 | 5 | -0.57 | -2.86 | -1.04 |
| 54 | 192903 | 193001 | 10 | -0.85 | -8.53 | -1.03 |
| 55 | 193312 | 193410 | 10 | -1.56 | -15.61 | -2.25 |
| 56 | 193610 | 193702 | 4 | -0.89 | -3.57 | -1.43 |
| 57 | 193802 | 193810 | 8 | -0.50 | -3.97 | -1.15 |
| 58 | 194001 | 194102 | 13 | -1.28 | -16.63 | -1.89 |
| 59 | 194111 | 194210 | 11 | -1.03 | -11.34 | -1.69 |
| 60 | 194410 | 194002 | 2 | -0.77 | -1.53 | -1.04 |
| 61 | 194605 | 194704 | 11 | -0.68 | -7.49 | -1.20 |
| 62 | 195212 | 195402 | 14 | -1.27 | -17.83 | -1.97 |
| 63 | 195510 | 195701 | 15 | -1.44 | -21.58 | -2.40 |
| 64 | 195902 | 196008 | 18 | -1.21 | -21.84 | -3.16 |
| 65 | 196301 | 196510 | 33 | -0.87 | -28.74 | -1.85 |
| 66 | 196903 | 197010 | 19 | -1.52 | -28.89 | -2.48 |
| 67 | 197109 | 197412 | 39 | -1.42 | -55.36 | -2.75 |
| 68 | 197512 | 197801 | 25 | -1.20 | -29.97 | -2.14 |
| 69 | 197902 | 197908 | 6 | -0.74 | -4.47 | -1.27 |
| 70 | 198502 | 198507 | 5 | -0.63 | -3.17 | -1.07 |
| 71 | 199603 | 199703 | 12 | -0.96 | -11.47 | -1.70 |

**Table S8 As Table S3 but for reconstructed Scot SPI-12.**

| **ID** | **Start Year/Month** | **End Year/Month** | **Duration (months)** | **Mean SPI-12** | **Accumulated SPI-12** | **Min SPI-12** |
| --- | --- | --- | --- | --- | --- | --- |
| 1 | 176211 | 176312 | 13 | -1.44 | -18.70 | -2.79 |
| 2 | 176502 | 176901 | 47 | -1.84 | -86.34 | -4.45 |
| 3 | 177208 | 177211 | 3 | -0.78 | -2.33 | -1.13 |
| 4 | 177708 | 177812 | 16 | -0.93 | -14.86 | -1.30 |
| 5 | 178002 | 178007 | 5 | -0.057 | -2.83 | -1.08 |
| 6 | 178012 | 178411 | 47 | -1.63 | -76.55 | -3.58 |
| 7 | 178805 | 178707 | 26 | -1.07 | -27.80 | -2.21 |
| 8 | 178812 | 178906 | 6 | -0.97 | -5.80 | -1.56 |
| 9 | 179503 | 179601 | 10 | -1.31 | -13.12 | -1.67 |
| 10 | 179701 | 179709 | 8 | -1.01 | -8.11 | -1.48 |
| 11 | 180308 | 180401 | 5 | -1.13 | -5.67 | -1.96 |
| 12 | 180510 | 180601 | 3 | -1.08 | -3.24 | -1.51 |
| 13 | 181212 | 181503 | 27 | -1.23 | -33.14 | -2.43 |
| 14 | 182003 | 182104 | 13 | -0.76 | -9.90 | -1.39 |
| 15 | 182612 | 182802 | 14 | -1.23 | -17.22 | -2.17 |
| 16 | 182906 | 183101 | 19 | -1.25 | -23.80 | -2.73 |
| 17 | 183108 | 183112 | 4 | -1.02 | -4.08 | -1.76 |
| 18 | 183211 | 183312 | 13 | -0.80 | -10.36 | -1.48 |
| 19 | 183412 | 183604 | 16 | -1.03 | -16.52 | -1.89 |
| 20 | 183801 | 183909 | 20 | -1.01 | -20.26 | -2.18 |
| 21 | 184102 | 184405 | 3 | -0.47 | -1.42 | -1.12 |
| 22 | 184211 | 184310 | 11 | -0.61 | -6.69 | -1.29 |
| 23 | 184503 | 184512 | 9 | -1.05 | -9.48 | -1.38 |
| 24 | 185203 | 185211 | 8 | -0.84 | -6.76 | -1.32 |
| 25 | 185410 | 185903 | 53 | -1.27 | -67.41 | -2.15 |
| 26 | 186106 | 186109 | 3 | -0.50 | -1.51 | -1.00 |
| 27 | 186410 | 186602 | 16 | -1.73 | -27.61 | -2.57 |
| 28 | 187008 | 187206 | 22 | -1.09 | -24.06 | -2.14 |
| 29 | 187510 | 187612 | 14 | -0.94 | -13.12 | -1.86 |
| 30 | 187905 | 187908 | 3 | -0.64 | -1.93 | -1.21 |
| 31 | 187911 | 188201 | 26 | -1.39 | -36.22 | -2.96 |
| 32 | 188508 | 188608 | 12 | -0.56 | -6.75 | -1.05 |
| 33 | 188710 | 188902 | 16 | -1.10 | -17.55 | -2.05 |
| 34 | 189106 | 189110 | 4 | -1.07 | -4.26 | -1.44 |
| 35 | 189502 | 189603 | 13 | -1.06 | -13.79 | -1.61 |
| 36 | 190003 | 190012 | 9 | -0.81 | -7.31 | -1.22 |
| 37 | 190112 | 190302 | 14 | -0.91 | -12.72 | -1.24 |
| 38 | 190902 | 191002 | 12 | -0.86 | -10.30 | -1.46 |
| 39 | 191107 | 191203 | 8 | -1.14 | -9.11 | -2.03 |
| 40 | 191512 | 191610 | 10 | -0.84 | -8.41 | -1.24 |
| 41 | 191702 | 191802 | 12 | -1.08 | -12.95 | -1.89 |
| 42 | 192111 | 192207 | 8 | -0.81 | -6.46 | -1.20 |
| 43 | 192904 | 192912 | 8 | -1.14 | -9.11 | -1.41 |
| 44 | 193208 | 193210 | 2 | -0.72 | -1.44 | -1.08 |
| 45 | 193311 | 193412 | 13 | -1.63 | -21.21 | -2.22 |
| 46 | 193802 | 193811 | 9 | -1.05 | -9.43 | -1.46 |
| 47 | 193910 | 194108 | 22 | -1.72 | -37.75 | -3.08 |
| 48 | 194110 | 194302 | 16 | -1.73 | -27.62 | -2.64 |
| 49 | 194605 | 194609 | 4 | -1.0 | -4.33 | -1.44 |
| 50 | 194708 | 194810 | 14 | -1.42 | -19.91 | -2.22 |
| 51 | 195301 | 195406 | 17 | -0.83 | -14.06 | -1.69 |
| 52 | 195511 | 195702 | 15 | -1.10 | -16.46 | -1.69 |
| 53 | 195908 | 196008 | 12 | -0.81 | -9.73 | -2.15 |
| 54 | 196301 | 196311 | 10 | -1.22 | -12.22 | -1.83 |
| 55 | 196903 | 197008 | 17 | -0.83 | -14.04 | -1.63 |
| 56 | 197111 | 197409 | 34 | -1.15 | -39.24 | -1.87 |
| 57 | 197511 | 197708 | 21 | -1.44 | -30.32 | -2.90 |
| 58 | 198609 | 198611 | 2 | -0.66 | -1.33 | -1.04 |
| 59 | 199110 | 199211 | 13 | -1.06 | -13.74 | -1.61 |
| 60 | 199311 | 199403 | 4 | -0.62 | -2.47 | -1.12 |
| 61 | 199602 | 199801 | 23 | -1.34 | -30.80 | -2.42 |
